# Time Delayed Causal Gene Regulatory Network Inference with Hidden Common Causes

**DOI:** 10.1371/journal.pone.0138596

**Published:** 2015-09-22

**Authors:** Leung-Yau Lo, Man-Leung Wong, Kin-Hong Lee, Kwong-Sak Leung

**Affiliations:** 1 Department of Computer Science and Engineering, The Chinese University of Hong Kong, Hong Kong, Hong Kong; 2 Department of Computing and Decision Sciences, Lingnan University, Tuen Mun, Hong Kong; University Medicine Greifswald, GERMANY

## Abstract

Inferring the gene regulatory network (GRN) is crucial to understanding the working of the cell. Many computational methods attempt to infer the GRN from time series expression data, instead of through expensive and time-consuming experiments. However, existing methods make the convenient but unrealistic assumption of *causal sufficiency*, i.e. all the relevant factors in the causal network have been observed and there are no unobserved common cause. In principle, in the real world, it is impossible to be certain that all relevant factors or common causes have been observed, because some factors may not have been conceived of, and therefore are impossible to measure. In view of this, we have developed a novel algorithm named HCC-CLINDE to infer an GRN from time series data allowing the presence of hidden common cause(s). We assume there is a sparse causal graph (possibly with cycles) of interest, where the variables are continuous and each causal link has a delay (possibly more than one time step). A small but unknown number of variables are not observed. Each unobserved variable has only observed variables as children and parents, with at least two children, and the children are not linked to each other. Since it is difficult to obtain very long time series, our algorithm is also capable of utilizing multiple short time series, which is more realistic. To our knowledge, our algorithm is far less restrictive than previous works. We have performed extensive experiments using synthetic data on GRNs of size up to 100, with up to 10 hidden nodes. The results show that our algorithm can adequately recover the true causal GRN and is robust to slight deviation from Gaussian distribution in the error terms. We have also demonstrated the potential of our algorithm on small YEASTRACT subnetworks using limited real data.

## Introduction

Knowing the gene regulatory network (GRN) in the cell is crucial to understanding the working of the cell. In the cell, some proteins are transcription factors (TFs) which trigger or inhibit the transcription of other gene(s), which are then translated into proteins with a delay. These delays have been known to affect the network stability, or cause oscillations [1–4]. A simplistic view of the GRN is a directed network resulting from the complex causal interactions between genes, where each directed link is labeled with the delay. Since experimentally determining the regulatory targets of each TF is expensive and time-consuming, there have been many computational methods that attempt to utilize high-throughput microarray and RNA-seq gene expression data to infer the GRN. High-throughput technology such as microarray or RNA-seq allows the expression of thousands of genes to be measured at the same time, and allows time series expression data to be obtained when this is done for a number of time points.

Even though many GRN inference methods have been developed, to our knowledge, they all implicitly make the assumption of *causal sufficiency*, i.e. all the relevant factors in the causal network have been observed and there are no unobserved common cause. This assumption is convenient, but very unrealistic. For example, miRNAs were previously not thought to take important roles in gene regulation. In principle, in the real world, it is impossible to be certain that all relevant factors or common causes have been observed, because some factors may not have been conceived of, and therefore are impossible to measure.

### Gene Network Inference

There have been many GRN inference algorithms and models, with different levels of details, see [5, 6] for surveys of GRN modelling and [7] for survey on GRN inference algorithms for microarray expression data.

Due to the nature of GRN, most models of GRN could be described as a graph, where the vertices are the genes under consideration, and the edges represent the regulatory relationships. Different levels of details could be achieved by labeling the edges with extra information. In the simplest case, an undirected graph could be used, in which case only an association network of the genes is captured. ARACNE [8] infers undirected network using mutual information, but also uses Data Processing Inequality to try to eliminate indirect interactions. C3NET [9] first identify gene pairs with significant mutual information, then link each gene to the neighbor (if any) with highest mutual information, and output an undirected and conservative network, with no other explicit means of eliminating indirect effect.

But without direction in the edges, there is no causal interpretation. On the other hand, directed edges could be used, as in [10], which uses genetic algorithm to optimize a score based on partial correlation, estimated regulatory direction and effect, but the output edges are not labeled with time delays. Since the process of gene transcription and mRNA translation both take time, and non-negligible translational time delays have been observed [11, 12]. Moreover, RNA polymerase, a main working protein in transcription, has been observed to pause during transcription, adding a cumulative of 204–307s over a 2.3kb region [13]. It is known that these delays affect the network stability, or cause oscillations [1–4]. Therefore, these delays should also be taken into account for a more accurate GRN model.

Some algorithms consider delay of only one time step, as in [14], which considers discretized expression data, and uses association rule mining to find frequent regulatory patterns, but without eliminating indirection association. Boolean network, e.g. in [15], is a classic model of GRN where the expression of each gene is discretized to only “on” or “off”, and the expression of each gene at the next time step is a boolean function of expression of its regulators at the current time step. Another popular class of GRN model is Ordinary Differential Equations (ODE), where the rate of change of expression of each gene is a (linear or nonlinear) function of the expression of the gene and its regulators. When discretized in time, it reduces to one time step model. Examples are [16], which uses Gaussian process for Bayesian inference of an ODE model; and DELDBN [17], which combines ODE model with local Bayesian analysis, and uses estimated Markov blanket as the regulators of each gene. There are also Dynamic Bayesian Network (DBN) based models, which avoids the limitation of plain Bayesian network that no cycles are allowed. An example is [18], which utilizes Bayesian structural expectation maximization to infer a one time step DBN model.

There are relatively few algorithms that infer multiple time delays. [19] first estimates the possible delays from pairwise mutual information from discretized expression data, then infer multiple time step DBN by minimizing MDL score using genetic algorithm. Banjo [20] also optimizes a score metric on DBN using discretized expression data by MCMC based method, and the program later allows multiple delay. TD-ARACNE [21] is an extension of ARACNE with time delays. But these algorithms do not label the edges of GRN with regulatory effect. In contrast, in DD-lasso [22], the expression of a gene is a linear combination of expression of its regulators at (possibly different) previous time steps. It first estimates the delays between each gene pairs by maximum likelihood, then uses lasso [23] to remove indirect effects and estimate the coefficients, therefore the edges are labeled with the delays as well as the regulatory effect. CLINDE [24] uses a similar model, but uses conditional independence of the shifted time series to estimate the delays and eliminate indirect effects.

Some other algorithms use perturbation data, or use a combination of perturbation data and time series expression. [25] is a parallel implementation of the Network Identification by multiple Regression (NIR) algorithm utilizing perturbation data. [26] needs promoter sequence and TF binding site information in addition to (non-time series) expression data. [27] is an Inductive Causation (IC) [28, 29] based method, which uses steady state data, with partial prior knowledge of ordering of regulatory relationship, and uses entropy to test conditional independence, giving an acyclic network where some edges may remain undirected. [30] uses convex programming on an ODE model using perturbation data. TSNI [31] solves a discretized linear ODE model using time series expression data after each gene is perturbed. [32] uses a Dynamic Nested Effects Model using perturbation data, where the delays are assumed to have exponential distribution. [33] uses knockdown data for Boolean network with exponentially distributed time delays.

### Causality Inference

Outside of GRN inference, there have been quite a lot of works in the last two decades on inferring a causal network from observational data. Under the framework, the unknown causal relationships are represented by a directed graph, and the observed correlations and partial correlations are related to *d-separations* among the variables (through statistical tests), and therefore constrain the possible structures of the graph. In the basic framework, correlations are used to give possible causal links (without direction), and partial correlations are used to remove indirect links, finally the assumption of *acyclicity* gives rise to orientation rules to give directions to the links. Therefore, sometimes some links would remain undirected, as both directions are possible. These researches have been well summarized by [28] and [29]. Most works focused on causal structures represented as directed acyclic graph (DAG), because time delays are not considered, and the data used are not time series data. [34] attempted to extend the method for acyclic graph to cyclic graph, with more complex orientation rules. More recently, LiNGAM [35] formulated the causality inference as Independent Component Analysis (ICA) for the linear non-Gaussian acyclic structural equation model, and [36] extended it to the cyclic case. [37] extended LiNGAM to add time delays by first fitting an autoregressive model for the time delayed effects, and then fit the residuals with LiNGAM. [38] revisited the concept of Granger Causality. [39] generalized Bayesian network structure learning to cyclic structure.

### Hidden Common Cause

**Fig 1:**
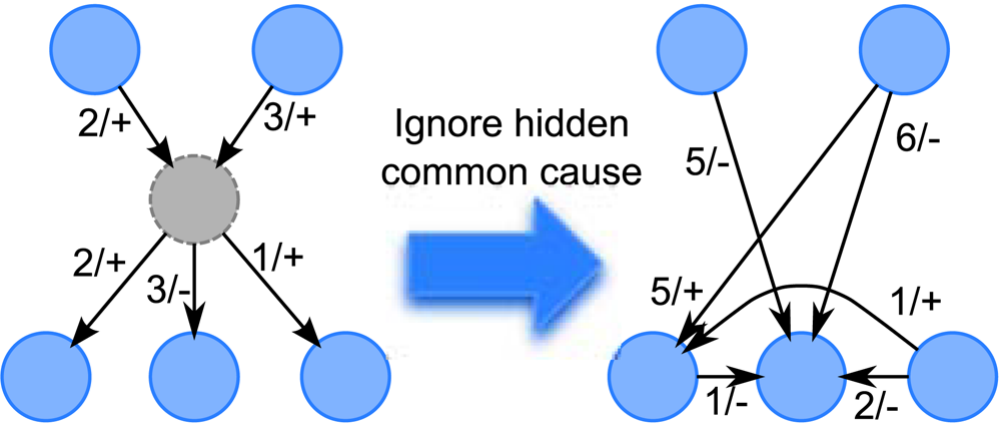
Example of Misleading Inference if Hidden Common Cause is Ignored. The number on the link is the delay, and + or − is the sign of the effect.

Hidden (latent) variables have been an important topic in causality inference. The problem is that when hidden common causes are ignored, the causality inferred could be misleading. This is illustrated in Fig 1, where some nodes are wrongly thought to be causally linked.

[40] is an early work that formulates the problem as determining the constraints on the variance-covariance matrix of observed data, then searching for a causal structure that would explain the constraints. Some works assume the presence of hidden common cause of observed variables, but focus only on the relationship of observed variables, with indication that some may have hidden common cause. For example, [41] is a Granger-causality based method that learns a mixed graph from time series data, where directed edges represent direct causal relationship, and dashed edges represent relationship due to hidden common cause. [42] is an extension of the FCI algorithm [29] and also outputs a mixed graph, but does not need time series data. [43] is another extension of the FCI algorithm, and learns an acyclic network with no time delays, and with no consideration that the latent variables may have observed variables as parents. In [44], a stochastic differential equation model (discretized in time) is used, where hidden variables are assumed, but only to more accurately estimate the relationship between observed variables, by a convex optimization based method. In [45], the d-separation and d-connection information, which are in practice provided by conditional tests, are encoded as a Satisfiability (SAT) problem, which is then incrementally solved to attempt to recover the dependency structure between observed variables, with indication that some have latent variables, but some edges may be marked as “unknown” if the given information cannot determine whether it is present or absent. [46] uses nested effects models using perturbation data with no time delays, and hidden common effect of two observed variables may be predicted, and some edges indicate possible presence of hidden nodes.

Some works assume that hidden variables may have other hidden variables as parents, but cannot have observed variables as parents. [47] and [48] are works in this direction, where observed variables are linear function of its parents (either hidden or observed), and hidden variables can be nonlinear function of its parents (only hidden variables). In [49], a linear Bayesian network is learnt, but it is assumed that there are no edges among observed variables, and that the hidden variables are linearly independent.

On the other hand, some works are more general and allow the hidden variables to have observed variables as parents. [50] uses a dynamic Bayesian network (DBN) model, and is based on the observation that in DBN, ignoring hidden variable usually results in violation of Markov property. Based on this, the algorithm tries to find non-markovian correlations (those across more than one time step) and introduce hidden variable to explain them. However, this work does not evaluate how close the resulting dependency structure is to the assumed causal structure, but only focuses on the likelihood on the testing set. [51] learns a discrete Bayesian network with hidden variable without time delays. It assumes that a hidden variable has observed variables as parents and children. It is motivated with the observation that when the hidden variable is not taken into account, the inferred dependency of the observed variables (the parents and children of the hidden variable) will usually be overly complicated, with many connections. Based on this, they find structural signature (semi-clique) to guess the position of the hidden variable(s) and its local structure, then make adjustment by explicitly linking the variables of the semi-clique with the introduced hidden variable. This local structure may then be fine-tuned by Structural-EM (SEM). This work also focuses on the likelihood in the evaluation, but not the inferred structure. This also places some restrictions on the subnetwork surrounding the hidden variable(s), e.g. a hidden variable must have parent(s), which are observed variable(s), also the total number of parents and children of a hidden variable must be at least four, as the smallest semi-clique has size four. [52] complements [51] and focuses on learning the dimensionality (the number of states) of hidden variables.

### Objective

First of all, it is important to emphasize that even inferring the causal relationships of observed variables is highly non-trivial, so inferring causal relationships of hidden variable(s) is obviously much more difficult and it is not possible to recover all possible cases of hidden variables. In some cases, the hidden variable is not very interesting, and in some cases, it would be too difficult to recover. For instance, if a hidden variable has no children, or one child with zero or one parent, it is not very interesting and is difficult to detect and estimate. The case that is feasible to handle is a hidden variable with two or more observed children, with or without parents, so we focus on this case.

In this paper, we assume that there is a sparse causal graph (possibly with cycles) of interest, where the variables are continuous and each causal link has a delay (possibly more than one time step). A small but unknown number of variables are not observed. Each unobserved (hidden) variable has only observed variables as children and parents, with at least two children and possibly no parents, and the children of unobserved variable(s) are not linked to each other. Although it is conceivable that two children of a hidden variable may be linked, so that a child has both the other child and the hidden variable as parents, it is difficult to differentiate whether the high correlation between two children are solely due to the hidden common cause, or also due to the presence of direct link. Therefore, we make the simplifying assumption that the children of hidden variable(s) are not linked to each other. Our objective is to infer the causal graph with the delays, given the time series of the observed variables. Since it is difficult to obtain very long time series, it is more realistic that the algorithm be also capable of utilizing multiple short time series, which we call *segments* in this paper. The segments are not necessarily of the same length (e.g. obtained from replicate experiments). To our knowledge, previous works either make much more restrictive assumptions or give different types of output (e.g. no delays in the links).

## Methods

**Fig 2:**
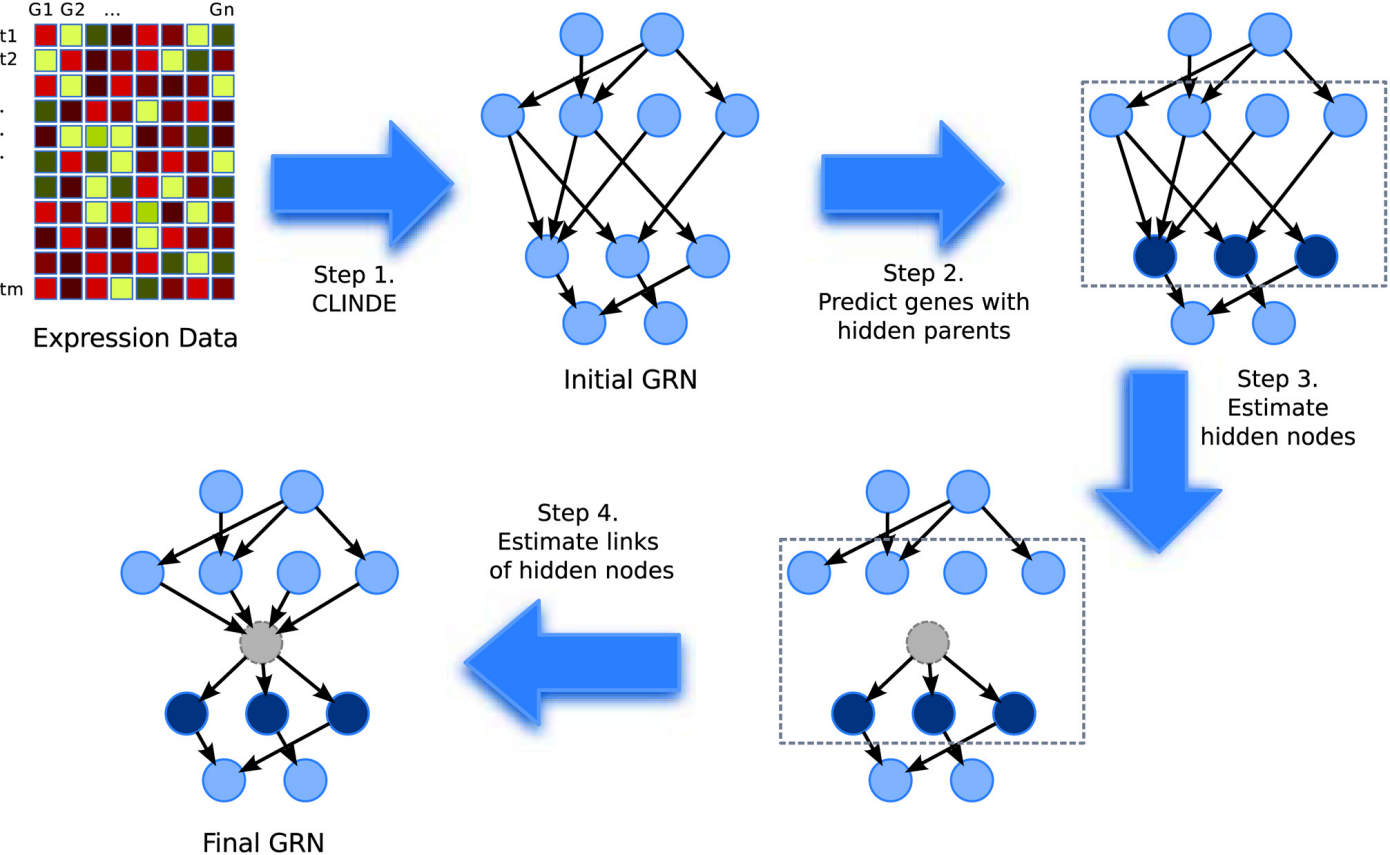
Overall Flow of the Algorithm. The steps are 1) infer an initial GRN of the observed genes, 2) determine the genes with hidden common cause(s) by the variance of the error terms, 3) estimate the hidden common cause(s), 4) infer the parents and children of the hidden common causes.

The overall flow of the proposed method is given in Fig 2. The steps are 1) infer an initial GRN of the observed genes, 2) determine the genes with hidden common cause(s) by the variance of the error terms, 3) estimate the hidden common cause(s), 4) infer the parents and children of the hidden common causes. The program code is written in C, any parameters described in this paper has a default value, and can be changed as needed. Also, sample running time is provided to give an idea of the running time needed. The program can be obtained at https://github.com/peter19852001/hcc_clinde. Below we first describe the data and model assumed in this paper, then describe each step in more details, where we first describe the case with one segment, and later we describe the case of multiple segments in a separate subsection.

### Data and Model

The GRN model assumed here is:

$$x_{j}\left(t\right)=\sum_{i=1}^{n+n_{h}}a_{ij}x_{i}\left(t-\tau_{ij}\right)+\epsilon_{j}\left(t\right)$$

where

- *x*_*i*_(*t*) is the expression value of gene *i* at time *t*, *i* = 1,…,*n* + *n*_*h*_, *t* = 1,…,*m*, and there are *n* observed genes, *n*_*h*_ hidden variable(s) and *m* equidistant time points.
- *n*_*h*_ is unknown, but $0\leq n_{h}<n$.
- *a*_*ij*_ is the regulatory effect of gene *i* on gene *j*, where the regulatory effect is repressive if *a*_*ij*_ is negative, activatory if positive, and absent if zero.
- *a*_*ij*_ = 0 for i,j>n, i.e. there are no causal links between hidden variables.
- for i>n, ∣{*j* : *a*_*ij*_ ≠ 0}∣ ≥ 2, i.e. each hidden variable has at least two observed genes as children.
- if a gene has a hidden parent, it has no other parents.
- *τ*_*ij*_ is the positive time delay of the edge *i* → *j* (if *a*_*ij*_ ≠ 0).
- *ϵ*_*j*_(*t*) is the error term for gene *j* at time *t*. We assume that E(*ϵ*_*j*_(*t*)) = 0 and Var(*ϵ*_*j*_(*t*)) = *σ* ^2^, i.e. the error terms are zero-mean and have fixed variance. They are also assumed to be *mutually independent*, but otherwise we do not make stringent assumptions on the distribution of the error terms.

Note that this model does not preclude self-regulation or cycles in the GRN, though any cycles must have positive delays. The given data is {*x*_*i*_(*t*)}, for *i* = 1,…,*n*. If the raw input data does not have equidistant time points, interpolation (e.g. spline interpolation) could be performed as preprocessing before using this algorithm.

### Initial GRN

The first step is to obtain an initial GRN. There are not many GRN inference methods that handles multiple time delays, CLINDE [24] and DD-lasso [22] are two of them, and both handles multiple short time series. We choose CLINDE to infer the initial GRN, based on the comparison in [24], which shows that CLINDE outperforms DD-lasso for smaller number of time points relative to the number of genes. Also because CLINDE does not restrict the multiple time series to be of the same length, unlike DD-lasso.

CLINDE is based on the PC algorithm [29], and consists of two stages. Stage 1 considers all (directed) pairs of genes *x* and *y*, and considers all possible delays *d* up to a maximum allowed delay, to determine if *x* → *y* is significant with the delay *d* based either on a correlation test, or mutual information test. The test is considered significant if the score of the test is larger than a score threshold. In the correlation test and partial correlation test below, the score is −log_10_(*p*-value), and in the mutual information test and conditional mutual information test below, the score is the (conditional) mutual information. So a higher score threshold means a more stringent test. And for correlation test, the regulatory effect (positive or negative) is also estimated if the edge is significant. After stage 1, there may be multiple edges from *x* to *y*, but with different delays. Also note that there may be cycles, but any cycle must have positive delays. Stage 2 attempts to prune the edges by partial correlation test or conditional mutual information test. Iteratively, the remaining edges are considered for pruning by conditioning first on *h* = 1 neighbor, then *h* = 2 neighbors, and so on up to *h* = *N*_0_, for a given parameter *N*_0_. In each iteration, each remaining edge is tested by conditioning on *h* neighbors, shifted properly using the delays estimated in stage 1. If the conditional test is not significant, the edge is pruned. After stage 2, the remaining edges are output as the GRN. Note that the pruning removes only indirect effects, and with sufficient data (so that the conditional tests truely reflects conditional independence relationships) any genuine cycles should remain, because each edge in the cycle is a direct effect. In this paper, we use (partial) correlation tests, which is the default for CLINDE. And after stage 2, the links with zero estimated delays are discarded to get the initial GRN for the following steps, as we assume the delays are positive.

Since CLINDE can infer cyclic GRN, so by using CLINDE for the initial GRN, HCC-CLINDE can handle cyclic GRN without further special handling.

### Identification and Estimation of Hidden Common Cause

After the initial GRN of the observed gene is obtained, for each gene, we can regress its expression on the shifted expression of its parents and obtain the corresponding error terms. Therefore we can calculate the empirical variance of the error terms. The idea is that for genes with hidden common causes, in the initial GRN, its parents would be determined incorrectly (likely the parent(s) or other children of the hidden variable) because the hidden variable is not observed, and consequently the variance of the error term is likely to differ from the expected fixed variance. On the other hand, if a gene has no hidden variable as parent, its parents would hopefully be determined correctly, and therefore the error term would have the expected variance. The variance of the error terms therefore provides a clue to whether a gene has hidden variable as parent.

**Fig 3:**
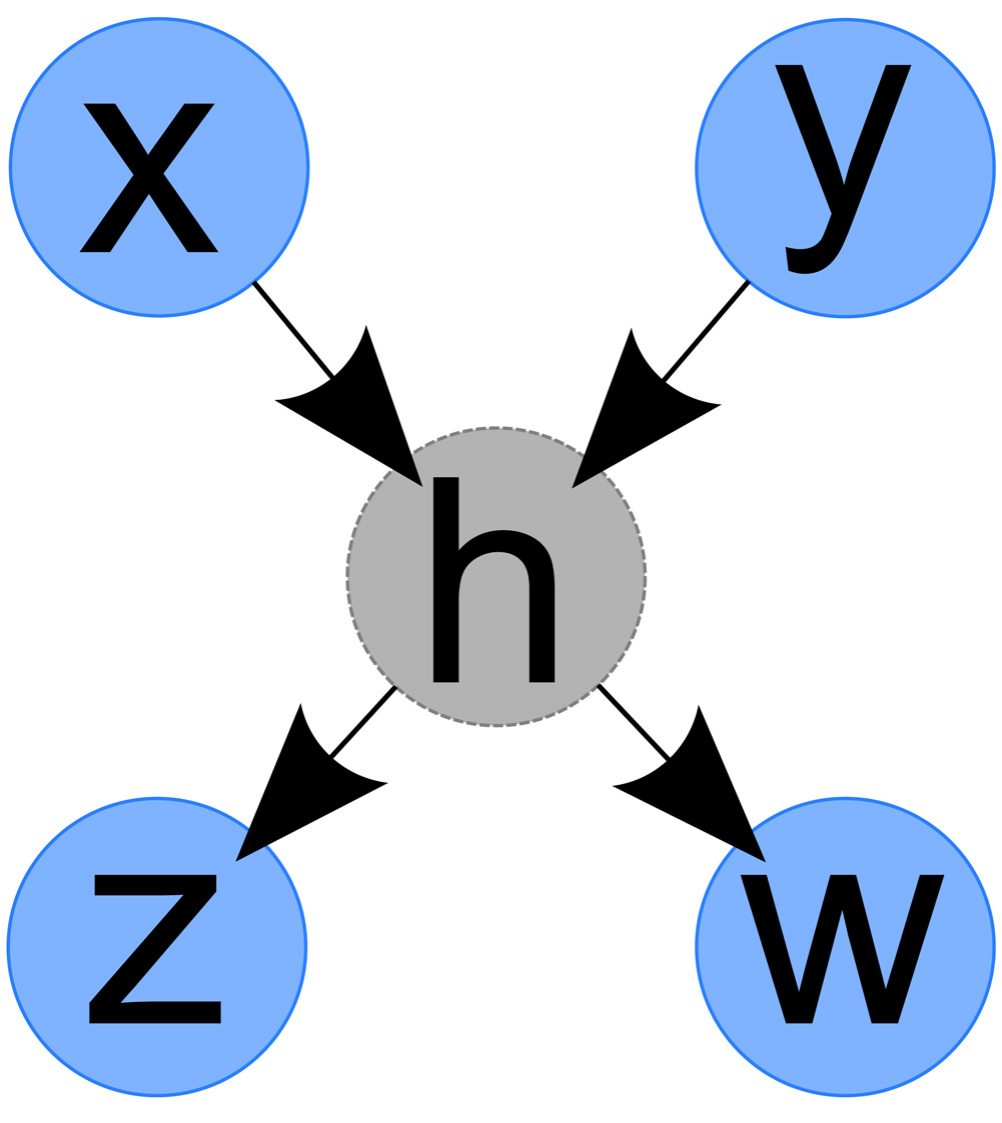
Example of Hidden Node. *X* and *Y* are independent. *H* is hidden.

To illustrate, consider the case depicted in Fig 3, where *X* and *Y* are independent and *H* is hidden:

$$\begin{array}{ccc} H & = & aX+bY+e_{h}\\ Z & = & cH+e_{z}\\ W & = & dH+e_{w} \end{array}$$

Suppose that in the initial GRN, the parents of *Z* are *X* and *Y*, so that *Z* = *c*(*aX* + *bY* + *e*_*h*_) + *e*_*z*_, and the variance of the error terms would be $\text{Var}\left(ce_{h}+e_{z}\right)=c^{2}\text{Var}\left(e_{h}\right)+\text{Var}\left(e_{z}\right)>\text{Var}\left(e_{z}\right)$. Similarly, if the parent of *Z* was incorrectly determined to be *W*, whereas we have $Z=\frac{c}{d}W-\frac{c}{d}e_{w}+e_{z}$, and the variance of the error terms would be $\text{Var}\left(\frac{c}{d}e_{w}+e_{z}\right)=\frac{c^{2}}{d^{2}}\text{Var}\left(e_{w}\right)+\text{Var}\left(e_{z}\right)>\text{Var}\left(e_{z}\right)$. In both cases, the variance of the fitted error terms would be larger than expected. On the other hand, in the initial GRN, if too many false parents were predicted for a gene, its error terms may have smaller variance than expected, but this is not common in CLINDE. In this paper, we use a simple thresholding to decide if a gene may have hidden parents: $\text{Var}\left(e\right)>\left(1+\rho \right)\sigma^{2}$, where Var(*e*) is the observed variance of the error terms of a gene, *ρ* ≥ 0 (with a default value of 0.1) is the tolerance, and *σ* ^2^ is the expected variance. Those genes determined to have hidden parents are called *candidates*.

If the number of observed genes *n* is small, we assume that the expected variance *σ* ^2^ is known and given. In contrast, if *n* is larger, we could instead estimate *σ* ^2^ from the observed variances of the error terms of the genes. For each observed gene, we can calculate the variance of the error terms based on the initial GRN. Based on the assumption that there are only a small number of hidden variables relative to the number of observed genes, in this paper we simply use the median of these variances as the estimate of the expected variance.

If there are no *candidates*, we output the initial GRN as the final GRN. Otherwise, we cluster them to estimate the hidden common cause for each cluster, based on the fact that genes with common parent are correlated. From our preliminary tests, we found that even a simple greedy clustering algorithm works adequately:

1. Let the *k* *candidates* be {*g*_1_,*g*_2_,…,*g*_*k*_}
2. Set *n*_*c*_ ← 1, *c*_1_ ← *g*_1_, *τ*_1_ ← 0, *C*_1_ ← {*g*_1_}
3. Set *ρ*_0_ ← (1+*ρ*)*e* ^2^, where *e* ^2^ is the expected variance of the error terms, and *ρ* is the tolerance
4. For *i* = 2,…,*k*
  - Let *d*_*i*_ = argmax_1 ≤ *j* ≤ *n*_*c*__ *d*(*c*_*j*_,*g*_*i*_), and set *τ*_*i*_ be the associated time shift of *g*_*i*_ relative to *c*_*j*_
  - If $d\left(c_{d_{i}},g_{i}\right)\geq \sqrt{\left(1-\frac{\rho_{0}}{\text{Var}(c_{d_{i}})})(1-\frac{\rho_{0}}{\text{Var}(g_{i})}\right)}$, update *C*_*d*_*i*__ ← *C*_*d*_*i*__∪{*g*_*i*_}
  - Otherwise, set *n*_*c*_ ← *n*_*c*_ + 1, then set *C*_*n*_*c*__ ← {*g*_*i*_}, *τ*_*i*_ ← 0
5. Output the *n*_*c*_ clusters {*C*_*j*_ : 1 ≤ *j* ≤ *n*_*c*_}, and the time shifts {*τ*_*i*_ : 1 ≤ *i* ≤ *k*}

where *c*_*i*_ is the center of cluster *i*, *C*_*i*_ is cluster *i*. *d*(*x*,*y*) measures the similarity of two time series *x* and *y*, here we use the maximum absolute correlation of the shifted time series (shift *y* relative to *x*, from −*τ*_0_ to *τ*_0_, where *τ*_0_ is the maximum delay).

The basic idea is that for each series, find out which cluster is the most similar to it, and if the similarity is high enough, it is included in that cluster; otherwise it becomes a new cluster. The similarity threshold is obtained from simple calculations as follows. Consider Fig 3 for *Z* and *W*, where *Z* = *cH* + *e*_*z*_ and *W* = *dH* + *e*_*w*_, so we have:

$$\begin{array}{ccc} \text{Var}(Z) & = & c^{2}\text{Var}(H)+\text{Var}(e_{z})=\frac{1}{1-\beta_{z}}c^{2}\text{Var}(H),\;\text{with}\;\beta_{z}=\frac{\text{Var}(e_{z})}{\text{Var}(Z)}\\ \text{Var}(W) & = & d^{2}\text{Var}(H)+\text{Var}(e_{w})=\frac{1}{1-\beta_{w}}d^{2}\text{Var}(H),\;\text{with}\;\beta_{w}=\frac{\text{Var}(e_{w})}{\text{Var}(W)}\\ \text{Corr}(Z,W) & = & \frac{\text{Cov}(Z,W)}{\sqrt{\text{Var}(Z)\text{Var}(W)}}=\frac{cd\text{Var}(H)}{\sqrt{\frac{1}{1-\beta_{z}}c^{2}\text{Var}(H)\frac{1}{1-\beta_{w}}d^{2}\text{Var}(H)}}\\ & = & \sqrt{\left(1-\beta_{z})(1-\beta_{w}\right)}\;\text{which decreases with increase in}\;\beta_{z}\;\text{and}\;\beta_{w} \end{array}$$

Since our threshold of the variance for *e*_*z*_ and *e*_*w*_ is *ρ*_0_ = (1 + *ρ*)*e* ^2^, for two genes *z* and *w* having a hidden common cause, we have the corresponding lower threshold of correlation as $\sqrt{\left(1-\frac{\rho_{0}}{\text{Var}(z)})(1-\frac{\rho_{0}}{\text{Var}(w)}\right)}$

**Fig 4:**
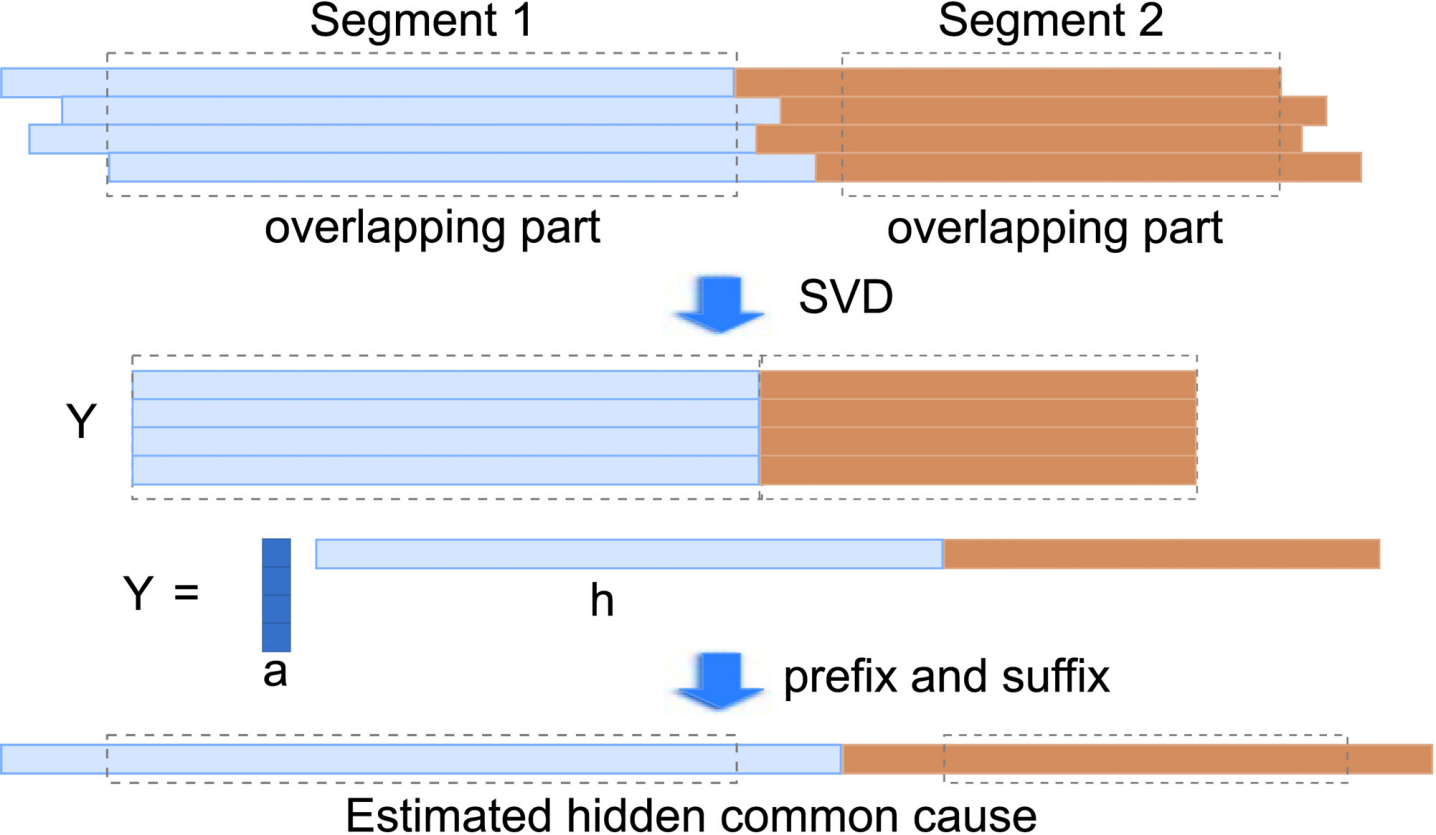
Illustration of Estimation of Hidden Common Cause from Un-aligned Time Series.

After the clustering, if there are no clusters with size larger than one, there is no need to estimate the hidden common causes, and we simply output the initial GRN as the final GRN. Otherwise, we would estimate a hidden common cause for each cluster *C*_*i*_ where $|C_{i}|>1$. Suppose *C*_*i*_ = {*g*_*i*,1_,*g*_*i*,2_…,*g*_*i*,∣*C*_*i*_∣_}, we first center the expression of these genes to get $x_{i,j}^{\prime }\left(t\right)=x_{i,j}\left(t\right)-\frac{1}{m}\sum_{\tau =1}^{m}x_{i,j}\left(\tau \right)$. Since each gene *g*_*i*,*j*_ has the associated time shift *τ*_*i*,*j*_ relative to the cluster center, we wish to estimate the coefficients $\vec{a_{i}}=\left[a_{i,1}\ldots a_{i,|C_{i}|}\right]$ and the hidden common cause *h*(*t*) where $x_{i,j}^{\prime }\left(t-\tau_{i,j}\right)\approx a_{i,j}h_{i}\left(t\right)$. Note that the scale of $\vec{a_{i}}$ and *h*(*t*) is undetermined as $a_{i,j}h_{i}\left(t\right)=\left(\frac{1}{\beta }a_{i,j}\right)\left(\beta h_{i}\left(t\right)\right)$ for any *β* ≠ 0. Also, since the time series may not be aligned (see Fig 4), so we first estimate the overlapping part, then estimate the prefix and suffix.

Suppose that relative to the cluster center, the overlapping parts are *t*_*s*_ ≤ *t* ≤ *t*_*e*_, let *Y*_*i*_ = [*y*_*kt*_] for 1 ≤ *k* ≤ ∣*C*_*i*_∣ and *t*_*s*_ ≤ *t* ≤ *t*_*e*_ and $y_{kt}=x_{i,k}^{\prime }\left(t-\tau_{i,k}\right)$, and let $\vec{h}=\left[h_{i}\left(t\right)\right]$ for *t*_*s*_ ≤ *t* ≤ *t*_*e*_, we want $Y_{i}\approx {\vec{a_{i}}}^{T}\vec{h}$, as illustrated in Fig 4. This is conveniently solved by Singular Value Decomposition of *Y*_*i*_ as low rank approximation to get $Y_{i}={\vec{u}}^{T}\sigma \vec{v}+E$ where *σ* is the largest singular value and the Frobenius norm of *E* is minimized. We then arbitrarily take $\vec{a_{i}}=\sigma \vec{u}$ and $\vec{h}=\vec{v}$. Having estimated the coefficients $\vec{a_{i}}$, we still need to estimate the non-overlapping prefix ($t<t_{s}$) and suffix ($t>t_{e}$) of *h*_*i*_(*t*), if any. For $t<t_{s}$ or $t>t_{e}$, we estimate *h*_*i*_(*t*) by minimizing ${\sum_{k}\left(a_{i,k}h_{i}\left(t\right)-x_{i,k}^{\prime }\left(t-\tau_{i,k}\right)\right)}^{2}$ where the sum is over *k* for which 1 ≤ *t*−*τ*_*i*,*k*_ ≤ *m*. This is readily solved to be $h_{i}\left(t\right)=\frac{\sum_{k}a_{i,k}x_{i,k}^{\prime }\left(t-\tau_{i,k}\right)}{\sum_{k}a_{i,k}^{2}}$.

Lastly, we take $h_{i}^{\prime }\left(t\right)=h_{i}\left(t+\max_{1\leq k\leq |C_{i}|}\tau_{i,k}+1\right)$ for 1 ≤ *t* ≤ *m*−1 and $h_{i}^{\prime }\left(m\right)=0$ as the estimate of the hidden common cause of cluster *C*_*i*_, i.e. take the suffix of *h*_*i*_(*t*) and shift by 1 so that $h_{i}^{\prime }\left(t\right)$ precedes all the genes in *C*_*i*_ in time.

### Inferring Causal Relationships of Estimated Hidden Common Cause

After estimating the hidden common cause(s), we attempt to re-estimate the subnetwork of the introduced hidden common cause(s), the *candidates*, and the parents of the *candidates*. For this, we apply CLINDE, with the restriction that in stage 1 of CLINDE, we do not consider the links in-between the *candidates* and links into the parents of the *candidates*, and consequently the resulting subnetwork will only consists of links from parents of *candidates* to hidden common causes and *candidates*, and from hidden common causes to *candidates*. Having obtained the subnetwork, we remove the links into the *candidates* in the initial GRN, and then union the resulting GRN with the subnetwork to get the final GRN. The rationale is that we expect the *candidates* to have hidden common causes as the real parents, where their apparent parents in the initial GRN may really be parents of the hidden common cause(s). Also, as the hidden common cause is estimated from the observed children, it is difficult to differentiate whether the high correlation between two children of a hidden variable is solely due to the hidden common cause, or that there is also a direct link between the two. In this paper, we therefore make the simplifying assumption that the children of a hidden variable are not linked to each other.

### Handling Multiple Segments of Time Series Data

**Fig 5:**
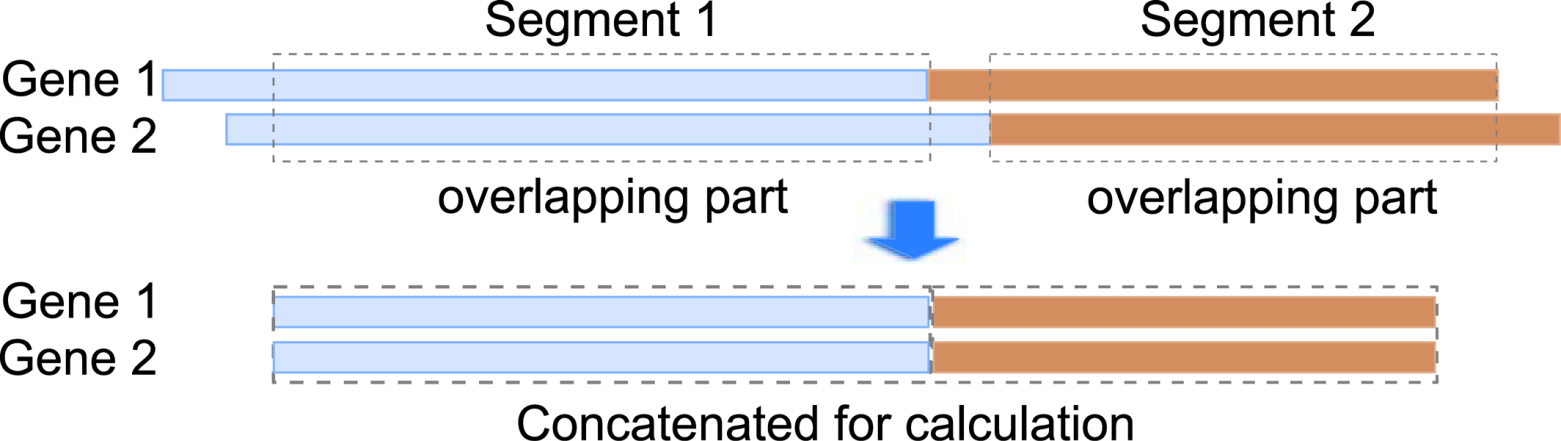
Illustration of Handling Multiple Segments of Time Series.

The above describes the steps of HCC-CLINDE when one segment of time series data is provided, we now consider the case where multiple segments of time series data are provided. We do not assume that the segments have the same lengths, but we assume that the time steps of different segments are the same. The main idea of handling multiple segments is that when the expression of genes have to be shifted by a delay (e.g. for calculating correlation), all the segments are shifted, and the overlapping parts are concatenated for the calculation. This is illustrated in Fig 5.

For inferring the initial GRN, we use CLINDE, which can handle multiple segments, in the same manner illustrated in Fig 5 for calculating correlation and partial correlation.

For identifying the candidate genes with hidden common cause, we need to first regress the expression of a gene on its parents (from the initial GRN), which is done by shifting multiple segments by the estimated delays using the overlapping parts for regression. After that, the clustering poses no difficulty as it only needs the correlation, which can be calculated in the same manner as in the initial GRN.

For estimating the hidden common cause(s) using SVD, we first shift and take the overlapping parts of the segments, and use SVD to estimate the center part of the hidden common cause for each segment and estimate the coefficients, then we go through each segment and estimate the prefix and suffix. This is illustrated in Fig 4.

Lastly, after the estimation of hidden common cause(s), we infer the causal links for the hidden common cause(s) by CLINDE on subnetwork, which poses no difficulty as CLINDE can handle multiple segments.

## Experiment Results

This section evaluates the effectiveness of our proposed algorithm HCC-CLINDE. We mainly rely on synthetic data, where we know the underlying gene network and the hidden variables, and the lack of sufficient expression data is not a concern. Since to our knowledge, there is no previous work that is similar to ours (infer hidden common cause with time delays), we can only compare our algorithm on incomplete data with CLINDE on incomplete and complete data, to show the improvement over ignoring hidden common cause.

We have tested on three types of synthetic data: 1) small GRN with one hidden node and the variance of error terms is known; 2) small GRN without hidden node and the variance of error terms is known; 3) large GRN with more than one hidden node and the variance of error terms is unknown. For all three cases, we try the score thresholds (st) 2, 3 and 4 for CLINDE, and use the default value for other CLINDE parameters. And for each case, we generate two types of data: one long segment of time series where we take prefixes of different lengths; and multiple segments where we use different number of segments for different total number of time points.

Due to the lack of long time series expression real data, we are unable to test our algorithm on large GRN, but we could demonstrate our algorithm on small GRNs. A dataset with relatively large number of time points is [53], which measures the expression of over 6,000 genes of Saccharomyces cerevisiae using DNA microarrays, with three different methods of synchronization for studying yeast cell cycle. Together with previous data from [54] (also included in [53]), we have 4 time series with information shown in Table 1. For the GRN, we use YEASTRACT [55], which is a curated database of over 200,000 transcription regulatory associations in Saccharomyces cerevisiae (including 113 TFs). Since the GRN is too large for the available expression data, we extract a small number of subnetworks for testing instead.

**Table 1:** Information of the Time Series Real Data.

**Series**	**Raw Time Points (Min)**	**Interpolated Time Points (Min)**
alpha	every 7 mins from 0 to 119	every 10 mins from 0 to 120
cdc15	10, 30, 50, 70, 80, 90, 100, 110, 120, 130, 140, 150, 160, 170, 180, 190, 200, 210, 220, 230, 240, 250, 270, 290	every 10 mins from 10 to 290
cdc28	every 10 mins from 0 to 160	same time points
elu	every 30 mins from 0 to 390	every 10 mins from 0 to 390

In the following, we first describe the performance metrics, and then the generation of synthetic expression data once the GRN is given, and then describe the generation of the synthetic GRN for different cases, and the results of the three types of synthetic data. After that, we describe the preprocessing of the YEASTRACT subnetworks and the expression data, and then present the results of our algorithm on the real data.

### Performance Metrics

It is more appropriate to focus on correctly predicting the presence of links rather than its absence, due to the sparse nature of GRNs. We assess the performance of the inference algorithm on three aspects, namely *Links* (which is considered correct if and only if both the gene pair and the direction are correct), *Delays* (which is considered correct if and only if both the link and the time delay *τ*_*ij*_ are correct) and *Effects* (which is considered correct if and only if both the link and the sign of the effect *a*_*ij*_ are correct). For each aspect, we look at three metrics respectively, namely *Recall* $=\frac{TP}{TP+FN}$, *Precision* $=\frac{TP}{TP+FP}$ and *F-score* $=\frac{2*Recall*Precision}{Recall+Precision}$, where *TP* is the number of *true positives*, *FP* is the number of *false positives*, *FN* is the number of *false negatives*. But since *F-score* is an overall measurement of performance, we focus on it.

**Fig 6:**
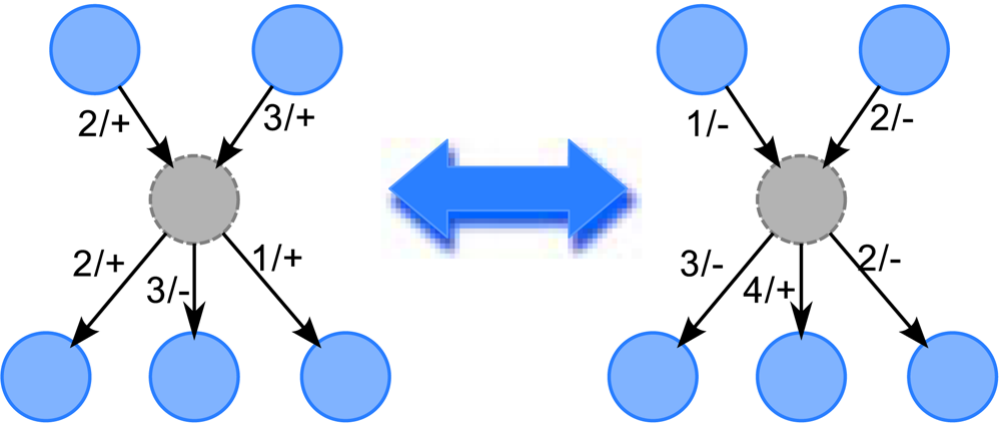
Example of Flipping Signs of Links and Shifting of Delays for Hidden Node. The number on the link is the delay, and + or − is the sign of the effect. Consistently flipping the signs and shifting the delays results in equivalent predicted GRN, as the hidden node is not observed.

We assume that in the true GRN, the hidden nodes are labeled. But in the predicted GRN, the number and the indices of the predicted hidden nodes may not be the same as the true GRN. Therefore, we need to map the predicted hidden nodes to the true GRN before doing the above performance calculations. Also, for the links to/from the hidden common nodes, the delays and the effects cannot be completely determined because there is no observed time series data for the hidden nodes to constrain them. This is illustrated in Fig 6, where the sign of a hidden node can be flipped, and be compensated by a flipping the signs of all its links; and the delays of links out of a hidden node can be shifted, and be compensated by the same shift of links into the hidden node. Therefore, in mapping the predicted hidden nodes to true hidden nodes, we may need to shift the delays, and flip the signs of the links appropriately, for useful calculation of the performance.

For each predicted hidden node, we try to align it to each of the true hidden nodes, and choose the one with the most matched links and delays (after shifting). And in case of ties, we arbitrarily choose true hidden node with the lowest index. When aligning a predicted hidden node, we consider only the links to/from observed genes, and consider all possible shifts in delay (for a shift of *d*, the delays in parent links will be increased by *d*, while that in children links will be decreased by *d*), and also whether to flip the signs of the links. How well it is aligned is measured by the sum of number of matched links and shifted delays. After the mapping of predicted hidden nodes (and shifting in delays and flipping of signs), the performance of the predicted GRN is calculated as described above.

### Generation of Synthetic Expression Data

#### One Segment

Given a network as (*a*_*ij*_,*τ*_*ij*_) where *a*_*ij*_ is the effect of gene *i* to gene *j*, and *τ*_*ij*_ is the associated delay if *a*_*ij*_ ≠ 0. Given the parameters *σ* ^2^ which is the variance of the error terms, and *α* which controls the gaussianity of error terms, we then generate the expression data with *m* time points as follows:

1. For $-\tau_{0}<t\leq 0$, 1 ≤ *j* ≤ *n*, set *x*_*j*_(*t*) = *e*_*j*_(*t*), where each *e*_*j*_(*t*) is generated from *N*(0,1)
2. For 1 ≤ *t* ≤ *m*, 1 ≤ *j* ≤ *n*, set $x_{j}\left(t\right)=\sum_{i=1}^{n}a_{ij}x_{i}\left(t-\tau_{ij}\right)+e_{j}\left(t\right)$, where *e*_*j*_(*t*) = sign(*z*_*jt*_)*s*∣*z*_*jt*_∣^*α*^, where each *z*_*jt*_ is generated from *N*(0,1) and *s* is used for scaling such that Var(*e*_*j*_(*t*)) = *σ* ^2^
3. Output {*x*_*j*_(*t*)}_1 ≤ *t* ≤ *m*,1 ≤ *j* ≤ *n*_

#### Multiple Segments

In order to generate *K* segments of time series data, we first randomly pick the lengths of each segment uniformly between 20 and 30 (inclusive), emulating the situation of multiple short time series, possibly with different lengths. Then we generate each segment of specified length as above.

### Synthetic Small GRN with One Hidden Node

We first test our proposed algorithm on small GRN where there is only one hidden node, and the variance of the error terms is assumed known.

#### Network Generation

**Fig 7:**
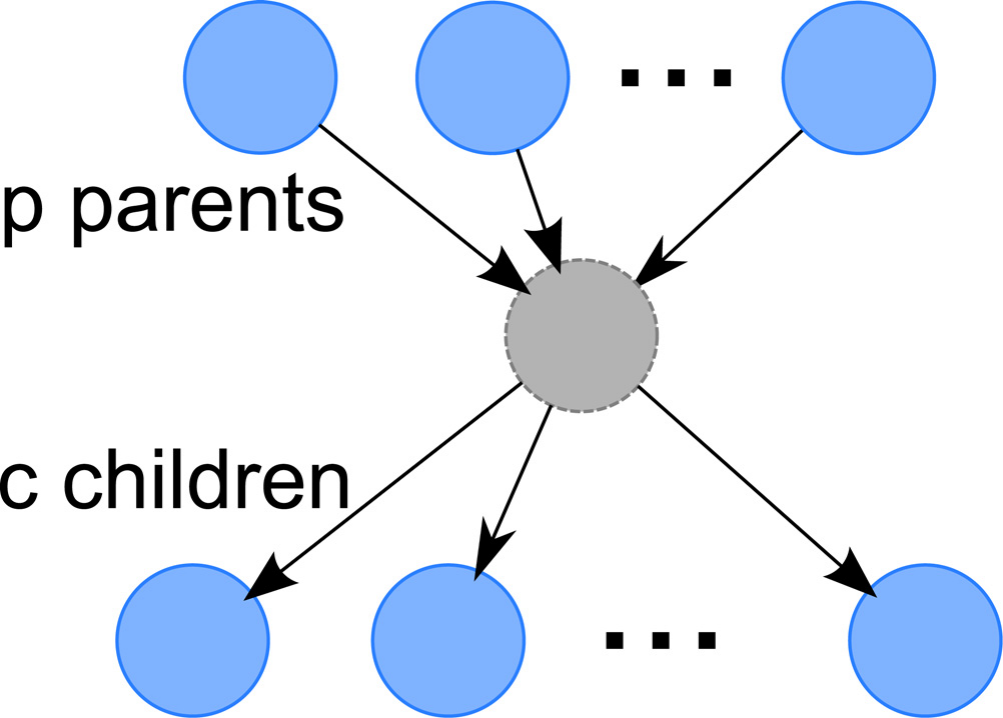
Small Synthetic GRN. There are *p* parents, *c* children and one hidden node.

In this small GRN case, each GRN has one hidden node, *p* observed parents and *c* observed children, as illustrated in Fig 7, but when applying the algorithm, we do not assume the presence of hidden node. For each link, the delay is uniformly chosen from {1,…,*τ*_0_}, where *τ*_0_ = 4; and the coefficient is *a*_*iu*_ = *ρ*_*iu*_ *z*_*iu*_, where *ρ*_*iu*_ is uniformly chosen from {−1,1} and *z*_*iu*_ is uniformly chosen from (0.5,1.5). Then we permute the indices of the observed genes.

We test a few values of the parameters, as shown in the column *Small GRN* of Table 2. For each setting of *p*, *c*, *σ* ^2^ and *α*, we generate 20 replicates, for a total of 6,400 GRNs. For the one segment case, for each replicate, we generate expression data with 200 time points, and then take prefix to get *m* time points, and output only the expression of the observed genes, for a total of 25,600 time series. And for the multiple segments case, for each replicate, we generate 8 segments, for a total of 51,200 segments, and for each setting, we test using *K* segments at a time.

**Table 2:** Parameter Settings of Synthetic Data Generation.

**Parameter**	**Small GRN**	**Large GRN**
Parents (*p*)	0, 1, 2, 3	—
Children (*c*)	2, 3, 4, 5	—
Observed genes (*n*)	*p* + *c*	50, 100
Hidden nodes (*n*_*h*_)	1 for hidden case, 0 for non-hidden case	5 for *n* = 50, 10 for *n* = 100
*σ* ^2^	0.5, 1, 2, 3, 4	0.5, 1, 2, 3, 4
*α*	0.5, 1, 2, 3	0.5, 1, 2, 3
Maximum parents (*M*_0_)	—	4
Maximum delay (*τ*_0_)	4	4
Replicates	20	40
Time points (*m*)	20, 50, 100, 200	20, 50, 100, 200, 400, 800
Number of segments (*K*)	1, 2, 4, 8	1, 2, 4, 8, 16, 32
*σ* ^2^ known?	Yes	No

#### Results

**Fig 8:**
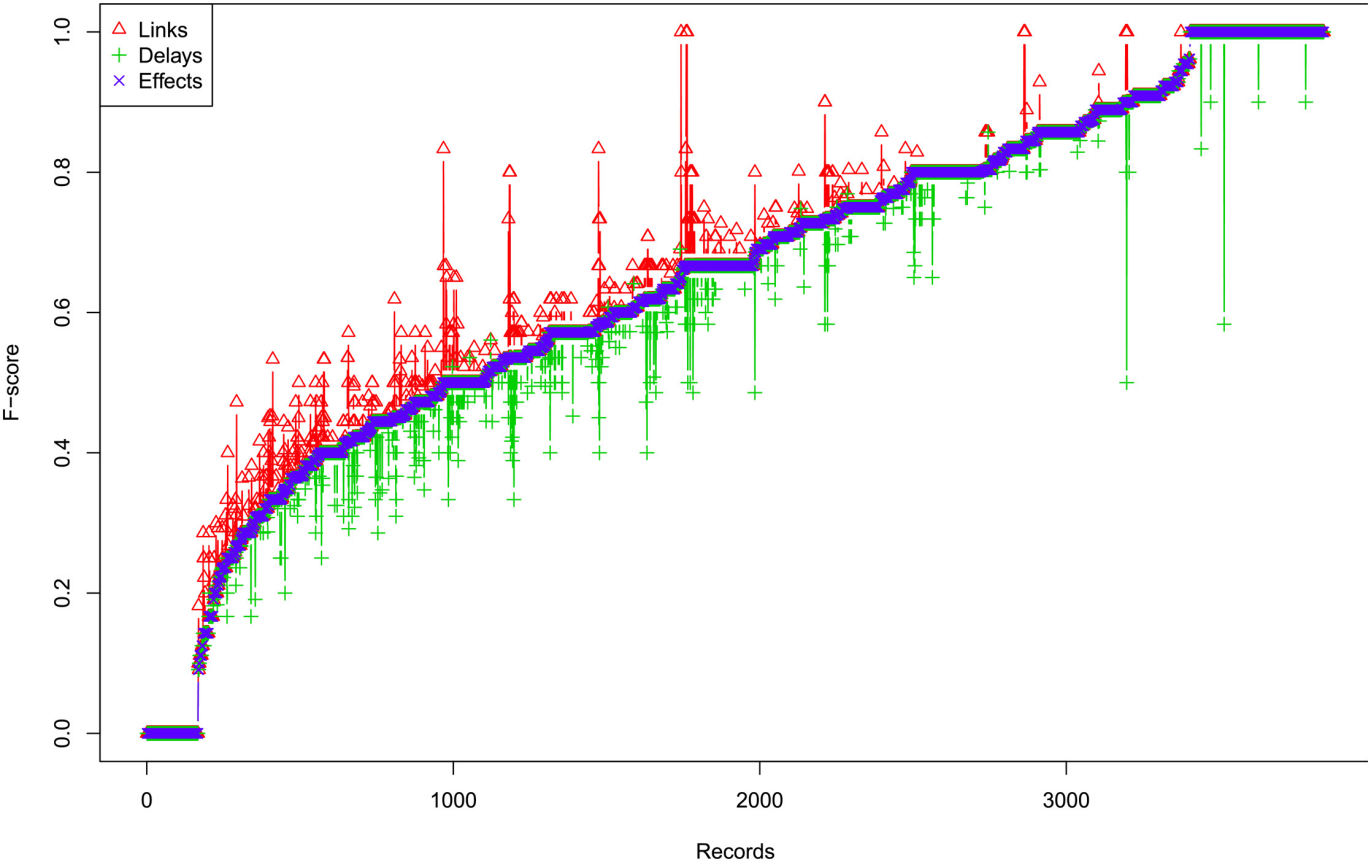
Profiles of F-scores of Links, Delays and Effects for Different Settings for Small Hidden Case. The x-axis shows the records.

**Fig 9:**
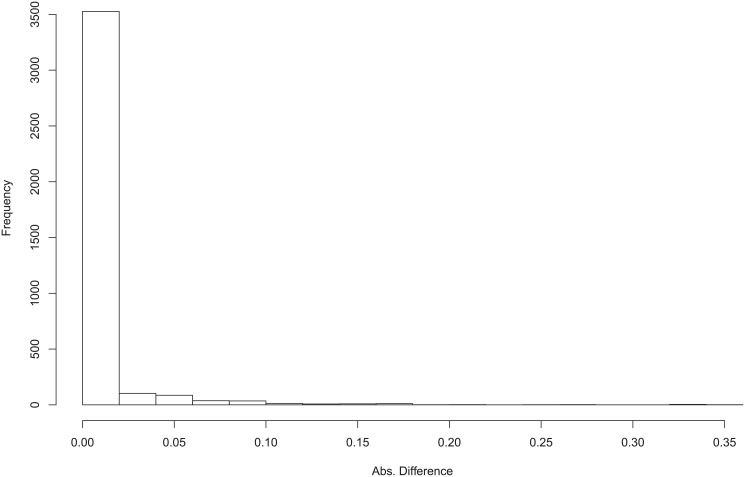
Histogram of Absolute Difference of F-scores of Links and Effects for Small Hidden Case.

First we show that the performances of *Links*, *Delays* and *Effects* are usually very consistent, with occasional discrepancies. Fig 8 shows the profiles of F-scores of *Links*, *Delays* and *Effects* for different settings for small hidden case with one segment time series. The three F-scores are mostly consistent with each other, though occasionally there are greater discrepancies for some records. In order to illustrate that these cases are very small, we plot the histogram of the absolute difference between *Links* and *Effects* F-scores in Fig 9. The histogram for difference of F-scores between *Delays* and *Effects* and between *Links* and *Effects* are similar and are shown in Figures A to B in S1 File. This suggests that the *Links*, *Delays* and *Effects* are usually inferred correctly at the same time, rather than getting one correct but the other incorrect. Therefore, in the following we mainly show the results of *Effects*, for the lack of space, but the full results including *Links* and *Delays* are provided in additional files.

**Fig 10:**
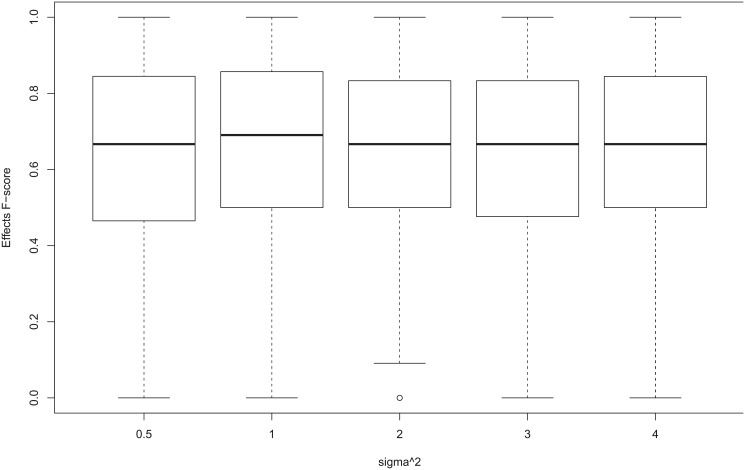
Boxplot of Effect F-score with Different *σ* ^2^ for Small Hidden Case.

Next we consider the F-scores under different *σ* ^2^. If we repeat the above method of showing the profiles of F-score for different *σ* ^2^, we found that the F-score of *individual record* for different *σ* ^2^ show great discrepancies for some records. However, Fig 10 shows the boxplot of *Effects* F-score for different *σ* ^2^ for one segment case, which shows that the overall distribution of F-scores are quite similar. The boxplot for the multiple segment case is similar and is shown in Figure L in S1 File. Therefore, we mainly show the results for *Effects* F-score for *σ* ^2^ = 2 in Table 3 for one segment case and in Table 4 for multiple segments case, and the full results of median performance for small hidden case are shown in Table A in S1 File for one segment case and in Table D in S1 File for multiple segment case.

**Table 3:** Median Effects F-scores for One Segment Small Hidden Case with *σ* ^2^ = 2.

			*α* = 0.5			*α* = 1			*α* = 2			*α* = 3		
p	c	*m*	st2	st3	st4	st2	st3	st4	st2	st3	st4	st2	st3	st4
0	2	20	0.667	0.667	0.667	0.500	0.500	0.583	0.000	0.000	0.000	0.000	0.000	0.000
		50	1.000	1.000	1.000	1.000	1.000	1.000	0.583	0.733	0.733	0.000	0.000	0.000
		100	1.000	1.000	1.000	1.000	1.000	1.000	1.000	1.000	1.000	0.000	0.000	0.000
		200	1.000	1.000	1.000	1.000	1.000	1.000	1.000	1.000	1.000	1.000	1.000	1.000
0	3	20	0.667	0.667	0.500	0.583	0.733	0.500	0.000	0.200	0.500	0.000	0.250	0.500
		50	0.833	0.900	0.800	1.000	1.000	1.000	0.733	0.800	0.800	0.450	0.536	0.536
		100	1.000	1.000	1.000	0.829	0.829	0.900	0.667	0.667	0.667	0.733	0.800	0.800
		200	1.000	1.000	1.000	1.000	1.000	1.000	1.000	1.000	1.000	1.000	1.000	1.000
0	4	20	0.500	0.619	0.667	0.619	0.619	0.667	0.571	0.667	0.667	0.143	0.333	0.367
		50	0.857	0.857	0.857	0.667	0.667	0.750	0.667	0.667	0.667	0.500	0.571	0.571
		100	0.750	0.804	0.857	0.750	0.857	0.857	0.708	0.750	0.804	0.708	0.708	0.708
		200	1.000	1.000	1.000	1.000	1.000	1.000	1.000	1.000	1.000	1.000	1.000	1.000
0	5	20	0.536	0.571	0.389	0.422	0.500	0.536	0.400	0.536	0.536	0.400	0.472	0.500
		50	0.764	0.800	0.844	0.727	0.750	0.844	0.633	0.664	0.727	0.400	0.400	0.586
		100	0.800	0.900	1.000	0.727	1.000	1.000	0.727	0.764	0.800	0.671	0.664	0.697
		200	1.000	1.000	1.000	0.955	1.000	1.000	1.000	1.000	1.000	0.955	1.000	1.000
1	2	20	0.400	0.400	0.500	0.400	0.400	0.650	0.536	0.667	0.500	0.000	0.000	0.000
		50	0.800	0.800	0.800	0.667	0.733	0.733	0.000	0.000	0.000	0.000	0.000	0.000
		100	1.000	0.900	0.900	0.800	0.800	0.800	0.667	0.733	0.733	0.143	0.167	0.167
		200	0.800	0.800	0.800	0.829	0.800	0.800	1.000	0.900	0.900	0.857	0.857	1.000
1	3	20	0.536	0.619	0.667	0.571	0.571	0.667	0.536	0.619	0.536	0.000	0.000	0.310
		50	0.873	0.873	0.857	0.661	0.750	0.708	0.708	0.667	0.667	0.571	0.571	0.571
		100	0.873	0.929	0.929	0.857	0.857	0.857	0.750	0.750	0.804	0.536	0.571	0.619
		200	1.000	1.000	1.000	0.857	0.857	0.857	0.857	0.857	0.857	0.857	0.857	0.857
1	4	20	0.667	0.667	0.571	0.522	0.500	0.500	0.545	0.667	0.667	0.191	0.216	0.250
		50	0.697	0.844	0.844	0.600	0.708	0.750	0.727	0.733	0.667	0.472	0.523	0.600
		100	0.909	1.000	1.000	0.800	0.800	0.844	0.844	0.844	0.889	0.545	0.600	0.633
		200	0.955	1.000	1.000	0.889	0.889	0.889	0.909	1.000	1.000	0.889	0.889	0.889
1	5	20	0.500	0.600	0.600	0.503	0.667	0.550	0.265	0.400	0.444	0.364	0.382	0.422
		50	0.748	0.800	0.800	0.697	0.748	0.727	0.667	0.667	0.667	0.445	0.523	0.481
		100	0.883	0.909	0.909	0.769	0.833	0.871	0.833	0.833	0.833	0.714	0.721	0.727
		200	0.909	0.909	0.909	0.962	1.000	1.000	0.923	0.909	0.909	0.871	0.909	0.909
2	2	20	0.310	0.333	0.367	0.000	0.143	0.619	0.250	0.286	0.367	0.000	0.292	0.367
		50	0.536	0.571	0.667	0.000	0.000	0.000	0.333	0.619	0.619	0.143	0.143	0.143
		100	0.571	0.667	0.619	0.571	0.571	0.619	0.667	0.667	0.667	0.417	0.417	0.452
		200	0.708	0.708	0.667	0.750	0.750	0.804	0.857	0.857	0.857	0.750	0.857	0.857
2	3	20	0.633	0.667	0.571	0.500	0.571	0.661	0.508	0.500	0.500	0.222	0.286	0.286
		50	0.667	0.708	0.708	0.573	0.522	0.583	0.444	0.472	0.500	0.000	0.111	0.450
		100	0.697	0.775	0.775	0.775	0.750	0.750	0.727	0.750	0.750	0.543	0.545	0.523
		200	0.817	0.889	0.889	0.775	0.775	0.775	0.800	0.889	0.889	0.800	0.889	0.844
2	4	20	0.545	0.633	0.600	0.396	0.545	0.453	0.091	0.091	0.291	0.321	0.364	0.382
		50	0.697	0.727	0.727	0.500	0.545	0.545	0.348	0.282	0.382	0.437	0.481	0.481
		100	0.833	0.833	0.833	0.633	0.748	0.748	0.606	0.606	0.606	0.641	0.586	0.633
		200	0.871	0.909	0.909	0.833	0.909	0.909	0.817	0.871	0.909	0.817	0.909	0.909
2	5	20	0.500	0.615	0.580	0.462	0.580	0.545	0.481	0.523	0.444	0.348	0.414	0.400
		50	0.667	0.641	0.667	0.708	0.769	0.742	0.571	0.593	0.580	0.268	0.297	0.321
		100	0.829	0.829	0.857	0.742	0.769	0.769	0.558	0.500	0.545	0.450	0.502	0.571
		200	0.857	0.923	0.923	0.862	0.890	0.923	0.857	0.923	0.923	0.785	0.857	0.857
3	2	20	0.347	0.500	0.500	0.250	0.286	0.333	0.286	0.286	0.310	0.000	0.111	0.268
		50	0.571	0.536	0.571	0.472	0.571	0.571	0.400	0.422	0.444	0.000	0.000	0.000
		100	0.500	0.571	0.571	0.523	0.571	0.536	0.472	0.417	0.417	0.100	0.000	0.000
		200	0.667	0.667	0.667	0.633	0.667	0.633	0.750	0.750	0.750	0.739	0.750	0.750
3	3	20	0.422	0.472	0.500	0.382	0.365	0.422	0.400	0.400	0.400	0.000	0.200	0.211
		50	0.586	0.573	0.600	0.382	0.422	0.422	0.573	0.600	0.600	0.400	0.400	0.422
		100	0.721	0.727	0.727	0.500	0.600	0.550	0.531	0.633	0.667	0.397	0.453	0.422
		200	0.769	0.800	0.764	0.721	0.727	0.727	0.817	0.800	0.800	0.769	0.800	0.764
3	4	20	0.414	0.481	0.523	0.348	0.364	0.400	0.382	0.422	0.400	0.321	0.297	0.279
		50	0.667	0.667	0.727	0.571	0.667	0.667	0.429	0.462	0.462	0.400	0.462	0.431
		100	0.714	0.690	0.690	0.742	0.769	0.769	0.593	0.615	0.615	0.438	0.462	0.413
		200	0.785	0.817	0.785	0.769	0.769	0.769	0.714	0.833	0.833	0.769	0.769	0.769
3	5	20	0.434	0.558	0.503	0.445	0.481	0.462	0.369	0.414	0.400	0.321	0.333	0.348
		50	0.646	0.667	0.667	0.625	0.593	0.641	0.471	0.571	0.536	0.388	0.388	0.429
		100	0.686	0.710	0.714	0.681	0.714	0.714	0.473	0.500	0.517	0.541	0.588	0.561
		200	0.750	0.800	0.800	0.840	0.866	0.857	0.866	0.866	0.866	0.800	0.875	0.866

The medians are taken over the 20 replicates. st2, st3 and st4 are for score thresholds of 2, 3 and 4 respectively.

**Table 4:** Median Effects F-scores for Multiple Segments Small Hidden Case with *σ* ^2^ = 2.

				*α* = 0.5			*α* = 1			*α* = 2			*α* = 3		
p	c	*K*	*m*	st2	st3	st4	st2	st3	st4	st2	st3	st4	st2	st3	st4
0	2	1	20	1.000	1.000	0.667	0.900	1.000	0.667	0.667	0.667	0.833	0.500	1.000	1.000
		2	43	1.000	1.000	1.000	1.000	1.000	1.000	0.900	1.000	1.000	0.500	0.500	0.833
		4	94	1.000	1.000	1.000	1.000	1.000	1.000	1.000	1.000	1.000	1.000	1.000	1.000
		8	201	1.000	1.000	1.000	1.000	1.000	1.000	1.000	1.000	1.000	1.000	1.000	1.000
0	3	1	30	0.667	0.800	0.800	0.800	0.800	0.800	0.733	0.800	0.800	0.667	0.800	0.800
		2	51	0.800	0.800	0.800	0.733	0.800	0.800	0.667	0.667	0.667	0.733	0.800	0.900
		4	100	0.829	0.800	0.800	1.000	1.000	1.000	0.833	0.800	0.800	0.800	0.733	0.800
		8	199	1.000	1.000	1.000	0.929	1.000	1.000	0.929	0.900	0.900	0.775	0.733	0.900
0	4	1	21	0.571	0.667	0.667	0.708	0.750	0.667	0.667	0.667	0.804	0.571	0.667	0.619
		2	48	0.750	0.750	0.857	0.857	0.857	0.857	0.750	0.750	0.804	0.750	0.750	0.857
		4	100	0.804	0.857	0.857	0.857	0.944	1.000	0.944	1.000	1.000	0.762	0.857	0.857
		8	204	0.889	1.000	1.000	1.000	1.000	1.000	1.000	1.000	1.000	0.889	0.929	0.929
0	5	1	25	0.495	0.619	0.571	0.633	0.633	0.571	0.550	0.633	0.750	0.545	0.739	0.750
		2	54	0.800	0.800	0.889	0.808	0.844	0.889	0.697	0.844	0.889	0.800	0.800	0.844
		4	110	0.764	0.800	0.844	0.817	0.889	0.944	0.727	0.844	0.889	0.800	0.800	0.889
		8	205	0.844	0.844	0.944	0.844	0.909	0.909	0.739	0.800	0.800	0.775	0.800	0.800
1	2	1	26	0.733	0.800	0.800	0.686	0.800	0.800	0.450	0.500	0.583	0.000	0.200	0.400
		2	51	0.800	0.800	0.800	0.800	0.800	0.800	0.333	0.367	0.400	0.167	0.367	0.367
		4	109	0.800	0.800	0.800	0.800	0.800	0.800	0.667	0.733	0.800	0.667	0.667	0.733
		8	195	0.800	0.800	0.800	0.733	0.800	0.800	0.667	0.733	0.800	0.333	0.286	0.333
1	3	1	23	0.750	0.762	0.667	0.619	0.667	0.667	0.675	0.804	0.857	0.667	0.571	0.667
		2	48	0.667	0.708	0.708	0.750	0.857	0.857	0.571	0.571	0.571	0.804	0.857	0.857
		4	96	0.750	0.750	0.750	0.857	0.857	0.857	0.804	0.857	0.857	0.804	0.857	0.857
		8	201	0.708	0.708	0.750	0.750	0.750	0.750	0.750	0.804	0.804	0.661	0.729	0.762
1	4	1	29	0.775	0.889	0.800	0.667	0.750	0.750	0.727	0.739	0.708	0.667	0.667	0.633
		2	50	0.844	0.844	0.889	0.708	0.708	0.708	0.733	0.733	0.708	0.633	0.667	0.708
		4	99	0.844	0.889	0.889	0.697	0.750	0.775	0.664	0.764	0.800	0.727	0.800	0.800
		8	191	0.800	0.889	0.844	0.800	0.889	0.844	0.800	0.800	0.764	0.664	0.800	0.800
1	5	1	28	0.817	0.833	0.800	0.727	0.667	0.697	0.748	0.800	0.800	0.727	0.697	0.697
		2	58	0.833	0.871	0.909	0.833	0.817	0.817	0.801	0.909	0.909	0.801	0.909	0.909
		4	105	0.909	0.909	0.909	0.909	0.909	0.909	0.839	0.877	0.909	0.785	0.833	0.833
		8	204	0.909	0.909	0.909	0.866	0.909	0.909	0.871	0.909	0.909	0.769	0.871	0.909
2	2	1	27	0.400	0.619	0.667	0.571	0.619	0.667	0.310	0.400	0.571	0.333	0.400	0.400
		2	54	0.667	0.667	0.667	0.000	0.571	0.571	0.571	0.619	0.667	0.667	0.667	0.667
		4	108	0.571	0.667	0.667	0.571	0.619	0.619	0.750	0.750	0.667	0.619	0.667	0.667
		8	216	0.667	0.667	0.667	0.619	0.619	0.619	0.750	0.750	0.750	0.708	0.708	0.667
2	3	1	21	0.586	0.619	0.571	0.472	0.571	0.571	0.500	0.571	0.500	0.444	0.500	0.472
		2	43	0.633	0.633	0.619	0.667	0.667	0.708	0.500	0.500	0.500	0.376	0.404	0.472
		4	98	0.600	0.586	0.586	0.550	0.600	0.600	0.422	0.472	0.472	0.522	0.556	0.556
		8	181	0.697	0.750	0.708	0.500	0.571	0.571	0.667	0.667	0.619	0.626	0.500	0.550
2	4	1	30	0.633	0.664	0.667	0.727	0.727	0.800	0.608	0.633	0.667	0.400	0.495	0.600
		2	58	0.641	0.667	0.697	0.748	0.800	0.800	0.641	0.697	0.727	0.748	0.727	0.727
		4	102	0.641	0.727	0.727	0.721	0.748	0.748	0.718	0.727	0.727	0.665	0.667	0.697
		8	209	0.630	0.665	0.690	0.667	0.785	0.800	0.748	0.727	0.727	0.665	0.697	0.697
2	5	1	22	0.615	0.667	0.667	0.615	0.586	0.573	0.445	0.500	0.600	0.286	0.354	0.422
		2	45	0.641	0.714	0.690	0.714	0.769	0.769	0.615	0.665	0.665	0.437	0.462	0.464
		4	93	0.690	0.714	0.769	0.690	0.714	0.714	0.769	0.769	0.769	0.354	0.414	0.381
		8	196	0.593	0.641	0.667	0.602	0.615	0.667	0.769	0.769	0.769	0.464	0.533	0.558
3	2	1	23	0.500	0.571	0.452	0.472	0.571	0.571	0.250	0.333	0.333	0.343	0.310	0.333
		2	50	0.500	0.500	0.571	0.500	0.536	0.571	0.250	0.250	0.268	0.404	0.310	0.393
		4	106	0.200	0.222	0.250	0.536	0.536	0.571	0.422	0.389	0.472	0.404	0.250	0.250
		8	202	0.422	0.444	0.444	0.500	0.500	0.500	0.200	0.286	0.286	0.536	0.500	0.536
3	3	1	28	0.450	0.600	0.633	0.545	0.633	0.667	0.573	0.600	0.500	0.382	0.400	0.444
		2	48	0.573	0.545	0.523	0.573	0.600	0.633	0.481	0.422	0.400	0.400	0.400	0.400
		4	98	0.586	0.600	0.600	0.396	0.523	0.473	0.545	0.573	0.600	0.472	0.573	0.500
		8	194	0.550	0.473	0.473	0.481	0.503	0.503	0.573	0.633	0.633	0.573	0.573	0.573
3	4	1	25	0.523	0.545	0.545	0.584	0.552	0.552	0.500	0.500	0.545	0.354	0.445	0.495
		2	53	0.464	0.473	0.473	0.500	0.523	0.545	0.536	0.558	0.580	0.429	0.462	0.500
		4	97	0.500	0.523	0.545	0.571	0.641	0.641	0.641	0.667	0.667	0.517	0.545	0.558
		8	188	0.533	0.558	0.580	0.431	0.462	0.545	0.571	0.598	0.620	0.433	0.466	0.481
3	5	1	23	0.517	0.593	0.593	0.533	0.571	0.593	0.598	0.615	0.615	0.450	0.466	0.462
		2	52	0.431	0.445	0.462	0.445	0.481	0.481	0.552	0.552	0.571	0.388	0.431	0.481
		4	105	0.588	0.620	0.641	0.590	0.690	0.714	0.620	0.690	0.769	0.556	0.641	0.615
		8	199	0.588	0.732	0.690	0.481	0.620	0.646	0.646	0.732	0.760	0.533	0.556	0.556

The medians are taken over the 20 replicates. st2, st3 and st4 are for score thresholds of 2, 3 and 4 respectively. *K* is the number of segments used. *m* is the total number of time points of the segments used.

The results of the small case with one hidden node have no clear trend for either score threshold, *α* or the number of time points *m*. We would expect that the performance is better with larger *m*, but there are some cases where this is not the case. One possible reason is that for this case, the algorithm need to correctly detect the presence of hidden common node, and infer the link(s) between the hidden node and the observed genes, so the performance has more variability. For example, if the algorithm incorrectly predicted that there are no hidden node, then the F-score will surely be 0, as in the true GRN, all links are to/from the hidden node. For *α*, there are some differences between *α* = 0.5,1 and 2, but the differences are not great. For *α* = 3, the algorithm seems to have worse performance when *m* is small. However, note that when *m* is large, the F-scores are quite satisfactory for one segment case, and in many settings are over 0.9 or even 1. For multiple segments case, for *p* = 2,3, the results are a bit worse, especially for *α* = 3, but can still reach 0.6 in F-score. The results show that the proposed algorithm can adequately detect and estimate the hidden node in a simple setting, and is robust towards slight deviation of gaussianity in the error terms.

### Synthetic Small GRN without Hidden Node

In this case, we test the effectiveness of the algorithm when there are in fact no hidden node. And in applying the algorithm, we do not assume the number of hidden nodes is known, but the variance of the error terms is known. The parameters are same as above, which is shown in the column *Small GRN* of Table 2.

#### Network Generation

For this purpose, we generate some “confusing” cases as follows. For each GRN (*p*, *c*, *σ* ^2^, *α* and replicate) in the above cases of small GRN with one hidden node, we use the (incomplete) data of 200 time points, and use CLINDE (with score threshold of 2 using PCor) to infer an GRN, which is definitely wrong, as CLINDE does not handle hidden nodes, and there are no true links among observed genes. If this GRN is non-empty, it is used; otherwise, a small GRN of a node in the middle with *p* parents, and *c*−1 children is generated as above, but all genes are labeled as observed. Having obtained the 6,400 GRNs without hidden nodes, the same method as above is used to generate the 25,600 time series data for one segment case, and 51,200 segments for multiple segments case.

#### Results

Similar to the small case with hidden node, the F-scores of *Links*, *Delays* and *Effects* are fairly consistent, so we omit the profiles and histograms here, but they are in Figures C to F in S1 File. Also, the situation is similar for the F-scores with different *σ* ^2^, and we show the boxplot in Figure J in S1 File for one segment case, and in Figure M in S1 File for multiple segments case. Therefore, we show the results of *Effects* for *σ* ^2^ = 2 in Table 5 for one segment case, and in Table 6 for multiple segments case, and the full results of median performance is shown in Table B in S1 File for one segment case and in Table E in S1 File for multiple segments case.

**Table 5:** Median Effects F-scores for One Segment Small Non-Hidden Case with *σ* ^2^ = 2.

			*α* = 0.5			*α* = 1			*α* = 2			*α* = 3		
p	c	*m*	st2	st3	st4	st2	st3	st4	st2	st3	st4	st2	st3	st4
0	2	20	0.000	0.000	0.000	0.000	0.000	0.000	0.000	0.000	0.000	0.500	0.000	0.000
		50	1.000	0.000	0.000	1.000	0.000	0.000	0.000	0.000	0.000	1.000	1.000	0.000
		100	1.000	1.000	1.000	1.000	1.000	1.000	1.000	1.000	0.000	0.333	0.000	0.000
		200	1.000	1.000	1.000	1.000	1.000	1.000	1.000	1.000	1.000	1.000	1.000	1.000
0	3	20	0.500	0.000	0.000	0.500	0.000	0.000	0.167	0.000	0.000	0.500	0.000	0.000
		50	0.733	0.619	0.000	0.667	0.667	0.000	0.667	0.167	0.000	0.583	0.000	0.000
		100	0.800	0.800	0.800	1.000	1.000	1.000	0.667	0.667	0.619	0.800	0.800	0.583
		200	1.000	1.000	0.900	1.000	1.000	1.000	1.000	1.000	1.000	1.000	1.000	1.000
0	4	20	0.333	0.000	0.000	0.000	0.000	0.000	0.333	0.000	0.000	0.333	0.333	0.143
		50	0.800	0.667	0.125	0.619	0.310	0.000	0.583	0.400	0.143	0.444	0.444	0.367
		100	0.857	0.829	0.800	0.750	0.750	0.667	0.667	0.667	0.523	0.667	0.536	0.536
		200	1.000	1.000	1.000	1.000	1.000	0.873	1.000	0.929	0.857	0.889	0.889	0.889
0	5	20	0.325	0.000	0.000	0.236	0.000	0.000	0.364	0.000	0.000	0.333	0.268	0.222
		50	0.571	0.500	0.364	0.500	0.422	0.292	0.481	0.367	0.254	0.500	0.472	0.348
		100	0.775	0.750	0.667	0.697	0.633	0.539	0.641	0.472	0.365	0.586	0.571	0.400
		200	0.916	0.916	0.804	0.857	0.845	0.800	0.873	0.873	0.857	0.899	0.909	0.909
1	2	20	0.450	0.000	0.000	0.500	0.000	0.000	0.000	0.000	0.000	0.400	0.450	0.250
		50	1.000	1.000	0.667	0.733	0.800	0.667	0.667	0.667	0.200	0.583	0.500	0.450
		100	1.000	1.000	1.000	1.000	1.000	1.000	1.000	1.000	1.000	0.733	0.667	0.667
		200	1.000	1.000	1.000	1.000	1.000	1.000	1.000	1.000	1.000	0.929	1.000	1.000
1	3	20	0.500	0.422	0.286	0.333	0.000	0.000	0.450	0.333	0.000	0.422	0.167	0.143
		50	0.800	0.775	0.633	0.775	0.619	0.333	0.523	0.400	0.310	0.571	0.500	0.500
		100	0.857	0.857	0.857	1.000	1.000	0.929	0.733	0.667	0.667	0.571	0.571	0.536
		200	0.889	0.944	0.889	1.000	1.000	1.000	1.000	0.944	0.857	1.000	1.000	0.929
1	4	20	0.236	0.000	0.000	0.211	0.211	0.000	0.422	0.100	0.111	0.348	0.400	0.268
		50	0.523	0.382	0.278	0.600	0.558	0.236	0.545	0.481	0.382	0.500	0.453	0.404
		100	0.800	0.775	0.775	0.800	0.817	0.750	0.739	0.667	0.558	0.608	0.641	0.593
		200	0.889	0.873	0.857	0.889	0.889	0.889	0.955	1.000	0.889	0.873	0.899	0.857
1	5	20	0.364	0.182	0.000	0.382	0.211	0.000	0.336	0.268	0.167	0.321	0.348	0.000
		50	0.552	0.382	0.321	0.620	0.500	0.364	0.438	0.364	0.364	0.552	0.545	0.523
		100	0.727	0.717	0.625	0.750	0.727	0.667	0.558	0.500	0.500	0.593	0.500	0.500
		200	0.845	0.916	0.697	0.916	0.899	0.829	0.883	0.909	0.732	0.833	0.857	0.829
2	2	20	0.000	0.000	0.000	0.583	0.167	0.000	0.000	0.000	0.000	0.400	0.310	0.000
		50	0.667	0.533	0.400	0.733	0.667	0.667	0.733	0.500	0.400	0.500	0.500	0.333
		100	0.829	0.929	0.667	1.000	1.000	0.900	0.829	0.929	0.800	0.708	0.800	0.775
		200	0.829	1.000	1.000	0.929	1.000	1.000	1.000	1.000	1.000	0.944	1.000	1.000
2	3	20	0.310	0.000	0.000	0.400	0.286	0.000	0.400	0.333	0.000	0.367	0.286	0.000
		50	0.697	0.667	0.472	0.697	0.667	0.571	0.667	0.523	0.367	0.667	0.586	0.472
		100	0.857	0.857	0.800	0.857	0.857	0.829	0.750	0.708	0.667	0.667	0.500	0.500
		200	0.899	0.909	0.857	0.873	0.916	0.944	0.873	0.944	0.889	0.889	0.889	0.857
2	4	20	0.404	0.111	0.000	0.500	0.348	0.000	0.236	0.250	0.100	0.286	0.308	0.222
		50	0.641	0.586	0.536	0.523	0.523	0.422	0.600	0.523	0.310	0.472	0.472	0.422
		100	0.861	0.845	0.750	0.667	0.667	0.646	0.633	0.633	0.633	0.667	0.602	0.561
		200	0.923	0.916	0.962	0.833	0.845	0.845	0.889	0.899	0.861	0.833	0.833	0.833
2	5	20	0.226	0.160	0.144	0.310	0.174	0.000	0.236	0.225	0.143	0.333	0.333	0.250
		50	0.615	0.429	0.358	0.523	0.523	0.400	0.517	0.517	0.410	0.563	0.481	0.333
		100	0.766	0.683	0.649	0.667	0.665	0.593	0.539	0.563	0.563	0.646	0.625	0.497
		200	0.857	0.845	0.775	0.875	0.857	0.845	0.833	0.714	0.667	0.840	0.812	0.769
3	2	20	0.333	0.000	0.000	0.000	0.000	0.000	0.310	0.000	0.000	0.268	0.000	0.000
		50	0.571	0.500	0.500	0.571	0.250	0.000	0.382	0.382	0.310	0.417	0.333	0.367
		100	0.667	0.800	0.733	0.667	0.667	0.667	0.633	0.571	0.586	0.500	0.400	0.333
		200	0.857	1.000	0.857	0.800	1.000	0.829	0.873	0.883	0.857	0.845	0.857	0.829
3	3	20	0.250	0.000	0.000	0.321	0.000	0.000	0.333	0.143	0.000	0.236	0.211	0.000
		50	0.633	0.545	0.389	0.500	0.523	0.422	0.472	0.444	0.444	0.414	0.431	0.400
		100	0.857	0.889	0.857	0.739	0.708	0.667	0.641	0.586	0.472	0.558	0.573	0.481
		200	0.909	1.000	0.899	0.889	0.889	0.889	0.873	0.889	0.889	0.817	0.829	0.775
3	4	20	0.388	0.100	0.000	0.268	0.222	0.000	0.236	0.286	0.202	0.321	0.236	0.222
		50	0.500	0.500	0.462	0.533	0.552	0.453	0.586	0.500	0.472	0.400	0.348	0.297
		100	0.727	0.697	0.608	0.739	0.727	0.633	0.641	0.608	0.545	0.608	0.523	0.500
		200	0.828	0.817	0.800	0.909	0.909	0.883	0.857	0.889	0.857	0.775	0.750	0.708
3	5	20	0.267	0.136	0.000	0.276	0.276	0.000	0.321	0.211	0.168	0.297	0.183	0.121
		50	0.500	0.364	0.258	0.471	0.343	0.286	0.462	0.353	0.286	0.466	0.437	0.354
		100	0.710	0.667	0.627	0.588	0.586	0.544	0.620	0.517	0.517	0.556	0.517	0.429
		200	0.812	0.857	0.812	0.866	0.812	0.667	0.824	0.838	0.718	0.800	0.806	0.750

The medians are taken over the 20 replicates. st2, st3 and st4 are for score thresholds of 2, 3 and 4 respectively.

**Table 6:** Median Effects F-scores for Multiple Segments Small Non-Hidden Case with *σ* ^2^ = 2.

				*α* = 0.5			*α* = 1			*α* = 2			*α* = 3		
p	c	*K*	*m*	st2	st3	st4	st2	st3	st4	st2	st3	st4	st2	st3	st4
0	2	1	28	1.000	0.000	0.000	0.333	0.000	0.000	1.000	0.000	0.000	0.333	0.000	0.000
		2	50	1.000	1.000	0.000	1.000	0.000	0.000	1.000	0.833	0.000	1.000	1.000	0.833
		4	110	1.000	1.000	1.000	1.000	1.000	1.000	1.000	1.000	1.000	1.000	1.000	1.000
		8	211	1.000	1.000	1.000	1.000	1.000	1.000	1.000	1.000	1.000	1.000	1.000	1.000
0	3	1	20	0.333	0.000	0.000	0.000	0.000	0.000	0.000	0.000	0.000	0.000	0.000	0.000
		2	43	0.619	0.417	0.333	0.667	0.000	0.000	0.667	0.000	0.000	0.667	0.667	0.583
		4	93	1.000	0.800	0.800	1.000	1.000	0.800	1.000	1.000	0.800	1.000	1.000	1.000
		8	197	1.000	1.000	1.000	1.000	1.000	1.000	1.000	1.000	1.000	1.000	1.000	1.000
0	4	1	20	0.364	0.000	0.000	0.343	0.000	0.000	0.000	0.000	0.000	0.286	0.000	0.000
		2	41	0.667	0.333	0.000	0.667	0.400	0.000	0.450	0.333	0.000	0.422	0.444	0.000
		4	89	1.000	0.857	0.667	0.829	0.667	0.667	0.667	0.571	0.523	0.762	0.733	0.500
		8	197	1.000	1.000	1.000	1.000	0.873	0.857	0.857	0.857	0.857	0.889	0.944	0.857
0	5	1	27	0.500	0.200	0.000	0.250	0.000	0.000	0.400	0.000	0.000	0.310	0.236	0.000
		2	51	0.641	0.500	0.268	0.667	0.333	0.182	0.558	0.500	0.222	0.545	0.382	0.222
		4	96	0.800	0.667	0.487	0.800	0.667	0.558	0.775	0.667	0.667	0.785	0.697	0.619
		8	194	1.000	0.962	0.873	0.833	0.857	0.817	0.889	0.857	0.857	0.857	0.899	0.844
1	2	1	21	0.667	0.667	0.000	0.333	0.000	0.000	0.000	0.000	0.000	0.400	0.000	0.000
		2	44	1.000	1.000	0.800	0.900	1.000	0.667	0.667	0.500	0.450	0.500	0.500	0.333
		4	94	1.000	1.000	1.000	1.000	1.000	1.000	1.000	1.000	1.000	0.733	0.667	0.667
		8	196	1.000	1.000	1.000	1.000	1.000	1.000	1.000	1.000	1.000	1.000	1.000	0.800
1	3	1	27	0.619	0.472	0.292	0.500	0.310	0.000	0.536	0.200	0.000	0.444	0.000	0.000
		2	54	0.800	0.800	0.800	0.800	0.800	0.667	0.600	0.619	0.536	0.571	0.571	0.500
		4	100	0.857	0.857	0.829	0.857	1.000	0.857	0.829	0.800	0.800	0.857	0.857	0.733
		8	211	0.889	0.873	0.873	1.000	1.000	1.000	0.857	0.929	0.857	1.000	1.000	0.857
1	4	1	29	0.586	0.348	0.000	0.500	0.382	0.000	0.500	0.293	0.091	0.382	0.211	0.000
		2	55	0.750	0.732	0.573	0.667	0.500	0.500	0.500	0.545	0.481	0.497	0.321	0.222
		4	97	0.775	0.775	0.750	0.775	0.697	0.667	0.857	0.775	0.697	0.697	0.586	0.481
		8	202	0.857	0.857	0.857	0.845	0.889	0.873	0.909	0.899	0.889	0.857	0.813	0.817
1	5	1	21	0.348	0.167	0.000	0.268	0.077	0.000	0.286	0.222	0.000	0.276	0.211	0.000
		2	44	0.517	0.400	0.301	0.445	0.400	0.286	0.445	0.310	0.367	0.500	0.453	0.388
		4	89	0.667	0.620	0.593	0.667	0.558	0.481	0.558	0.517	0.400	0.641	0.571	0.458
		8	187	0.845	0.727	0.727	0.873	0.882	0.690	0.833	0.732	0.646	0.800	0.833	0.764
2	2	1	20	0.400	0.000	0.000	0.400	0.000	0.000	0.310	0.000	0.000	0.333	0.000	0.000
		2	44	0.667	0.500	0.367	0.733	0.667	0.536	0.667	0.583	0.000	0.667	0.500	0.500
		4	96	0.829	0.800	0.667	1.000	1.000	0.800	0.750	0.733	0.667	0.857	1.000	0.900
		8	193	1.000	1.000	1.000	1.000	1.000	1.000	1.000	1.000	1.000	1.000	1.000	1.000
2	3	1	22	0.500	0.400	0.000	0.400	0.000	0.000	0.310	0.000	0.000	0.422	0.268	0.310
		2	48	0.750	0.667	0.472	0.800	0.775	0.583	0.536	0.400	0.367	0.500	0.444	0.444
		4	103	0.829	0.857	0.800	0.857	0.873	0.829	0.708	0.708	0.667	0.697	0.708	0.633
		8	197	0.889	0.889	0.857	0.857	0.889	0.873	0.857	0.800	0.800	0.800	0.800	0.739
2	4	1	28	0.500	0.268	0.000	0.382	0.367	0.367	0.422	0.348	0.250	0.437	0.343	0.200
		2	55	0.739	0.536	0.481	0.667	0.573	0.533	0.641	0.481	0.400	0.517	0.500	0.462
		4	102	0.817	0.800	0.739	0.764	0.748	0.671	0.714	0.667	0.608	0.567	0.533	0.481
		8	200	0.873	0.889	0.899	0.889	0.833	0.833	0.873	0.899	0.817	0.785	0.733	0.733
2	5	1	28	0.364	0.258	0.148	0.400	0.287	0.167	0.336	0.182	0.211	0.330	0.167	0.138
		2	58	0.600	0.466	0.429	0.611	0.500	0.481	0.502	0.402	0.336	0.517	0.517	0.445
		4	113	0.714	0.750	0.598	0.785	0.774	0.646	0.686	0.602	0.528	0.580	0.539	0.558
		8	216	0.875	0.866	0.768	0.817	0.857	0.817	0.828	0.817	0.775	0.706	0.706	0.667
3	2	1	20	0.143	0.000	0.000	0.111	0.000	0.000	0.111	0.000	0.000	0.111	0.000	0.000
		2	40	0.667	0.500	0.400	0.400	0.333	0.000	0.125	0.286	0.000	0.333	0.310	0.167
		4	91	0.857	0.929	0.667	0.708	0.667	0.500	0.708	0.500	0.472	0.571	0.550	0.500
		8	198	1.000	1.000	1.000	0.873	0.857	0.667	0.873	0.800	0.708	0.800	0.800	0.800
3	3	1	28	0.422	0.400	0.250	0.558	0.400	0.286	0.400	0.000	0.000	0.267	0.222	0.000
		2	49	0.727	0.667	0.400	0.571	0.523	0.444	0.444	0.444	0.250	0.382	0.348	0.333
		4	94	0.708	0.800	0.667	0.697	0.750	0.667	0.667	0.727	0.667	0.558	0.500	0.422
		8	188	0.845	1.000	0.889	0.873	0.857	0.817	0.857	0.889	0.750	0.739	0.721	0.667
3	4	1	22	0.333	0.222	0.111	0.348	0.250	0.000	0.333	0.208	0.000	0.286	0.111	0.000
		2	47	0.571	0.500	0.400	0.586	0.517	0.437	0.544	0.481	0.400	0.400	0.429	0.388
		4	105	0.769	0.667	0.594	0.748	0.727	0.667	0.721	0.764	0.608	0.615	0.544	0.466
		8	207	0.833	0.833	0.800	0.909	0.909	0.833	0.889	0.889	0.833	0.769	0.769	0.760
3	5	1	24	0.321	0.250	0.133	0.301	0.267	0.071	0.400	0.308	0.148	0.243	0.194	0.183
		2	47	0.500	0.471	0.381	0.453	0.321	0.297	0.375	0.429	0.286	0.429	0.348	0.297
		4	93	0.690	0.571	0.541	0.615	0.485	0.402	0.717	0.578	0.513	0.533	0.502	0.450
		8	197	0.845	0.845	0.753	0.778	0.683	0.588	0.812	0.845	0.801	0.775	0.626	0.602

The medians are taken over the 20 replicates. st2, st3 and st4 are for score thresholds of 2, 3 and 4 respectively. *K* is the number of segments used. *m* is the total number of time points of the segments used.

For this case, when *m* is larger, the F-score is usually higher, although there are some exceptions, especially for *α* = 3 and *p* = 0. Also, small score thresholds usually work well, especially when *m* is small. The F-score is often 0 for small *m* with high score threshold, which means the score threshold is too stringent. When *m* is larger, using a more stringent score threshold also gives good F-score, but is only occasionally better than small score threshold. The F-scores can reach over 0.9 or even 1 in many settings, and over 0.6 in all settings. Similar to the above case, the performance does not show great difference for different *α*, though small *α* is usually slightly better. The results show that the proposed algorithm works satisfactorily even when there are no hidden node in a simple setting.

### Synthetic Large GRN with More than One Hidden Node

The above two cases are for small GRN, and where the variance of the error terms is known. We also test the more realistic case of larger GRN with more than one hidden node (but the number is unknown), and that the variance of the error terms is unknown, but estimated by the algorithm. For a network with *n* observed genes, we would generate $n_{h}=\left\lceil \frac{n}{10}\right\rceil$ hidden nodes.

#### Network Generation

**Fig 11:**
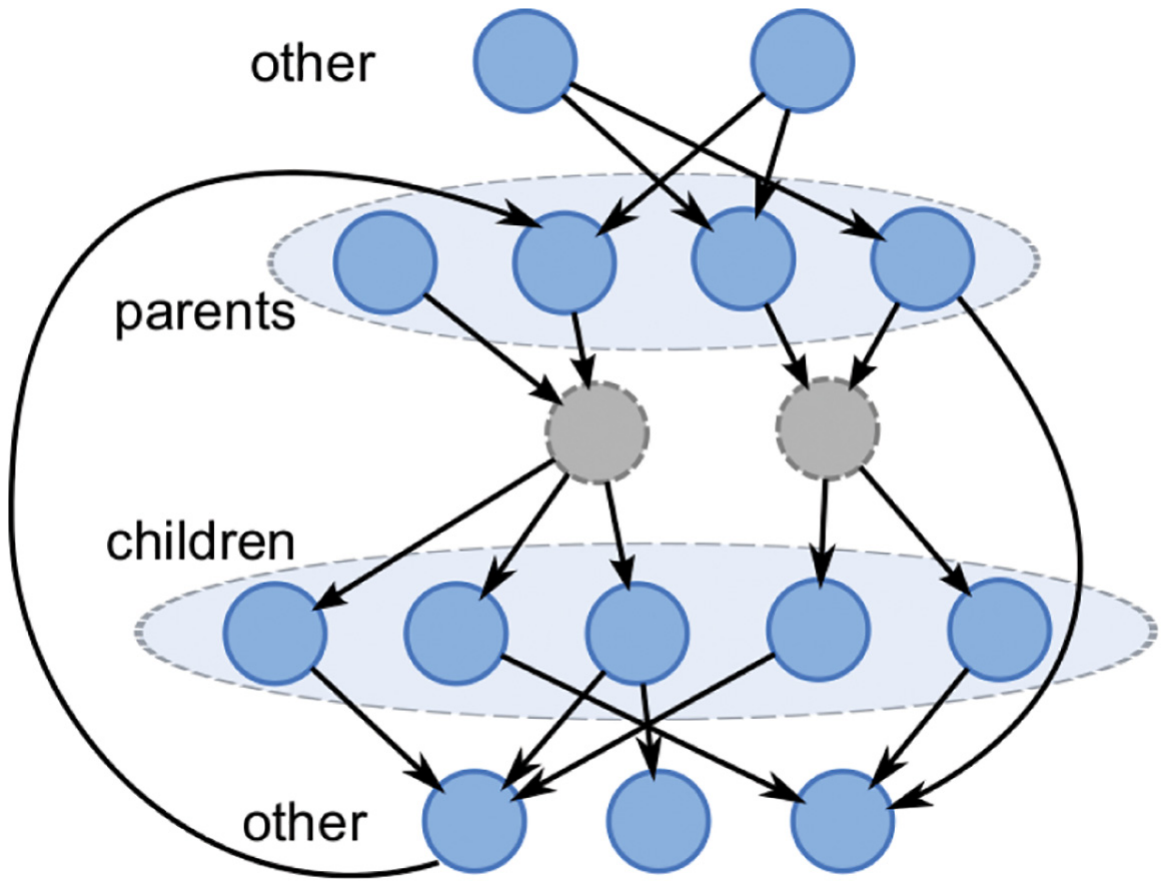
Large Synthetic GRN. Each hidden node has up to 3 distinct parents, and up to 5 distinct children.

For *n* observed genes and *n*_*h*_ hidden nodes, a maximum of *M*_0_ parents for observed genes, maximum of *τ*_0_ as delay, we generate a GRN with the structure shown in Fig 11 as follows:

1. Choose the number of parents (all distinct) for the hidden nodes: first for each *i* ∈ {0,1,2,3}, assign $\left\lfloor \frac{n_{h}}{4}\right\rfloor$ nodes to have *i* parents; for each of the remaining $n_{h}-\left\lfloor \frac{n_{h}}{4}\right\rfloor$ nodes, randomly choose *i* from {0,1,2,3} as the number of parents.
2. Similarly choose the number of children (all distinct and distinct from the parents) for the hidden nodes: first for each *i* ∈ {2,3,4,5}, assign $\left\lfloor \frac{n_{h}}{4}\right\rfloor$ nodes to have *i* children; for each of the remaining $n_{h}-\left\lfloor \frac{n_{h}}{4}\right\rfloor$ nodes, randomly choose *i* from {2,3,4,5} as the number of children.
3. Let *N*_*p*_ and *N*_*c*_ be the total number of parents and children of hidden nodes respectively.
4. Let *N*_*o*_ = *n*−*N*_*p*_−*N*_*c*_ (*n* should be large enough relative to *n*_*h*_ so that $N_{o}>0$)
5. For 1 ≤ *i* ≤ *N*_*o*_, randomly choose *p*_*i*_ from {0,…,*M*_0_}, and randomly link *p*_*i*_ parents from {1,…,*n*} to node *i*.
6. For $N_{o}<i\leq N_{o}+N_{p}$, randomly choose *p*_*i*_ from {0,…,*M*_0_}, and randomly link *p*_*i*_ parents from {1,…,*n*−*N*_*c*_} to node *i*.
7. For the hidden nodes, link from distinct parents from {*N*_*o*_ + 1,…,*N*_*o*_ + *N*_*p*_}, respecting the number of parents chosen above.
8. Similarly link to distinct children for the hidden nodes from {*N*_*o*_ + *N*_*p*_ + 1,…,*n*}, respecting the number of children chosen above.
9. For each link *L*, randomly set the coefficient as *a*_*L*_ = *ρ*_*L*_ *z*_*L*_, where *ρ*_*L*_ is uniformly chosen from {−1,1} and *z*_*L*_ is uniformly chosen from (0.5,1.5).
10. For each link *L*, uniformly choose *τ*_*L*_ from {1,…,*τ*_0_}.
11. Since the GRN may have cycles, scale the coefficients to make the GRN stable.
12. Lastly, randomly permute the indices of the observed genes {1,…,*n*}.

The parameters that we have tested are listed in column *Large GRN* of Table 2. For each setting of *n*, *σ* ^2^, *α*, we randomly generate 40 replicates as described above, for a total of 1,600 GRNs. Then for each GRN, for the one segment case, we generate expression data with *m* = 800 time points, and take prefixes to get different number of time points. So there are a total of 9,600 time series. And for the multiple segments case, we generate 32 segments for each replicate, for a total of 51,200 segments. We test more time points because now the number of genes is larger than the above two small cases.

#### Results

**Fig 12:**
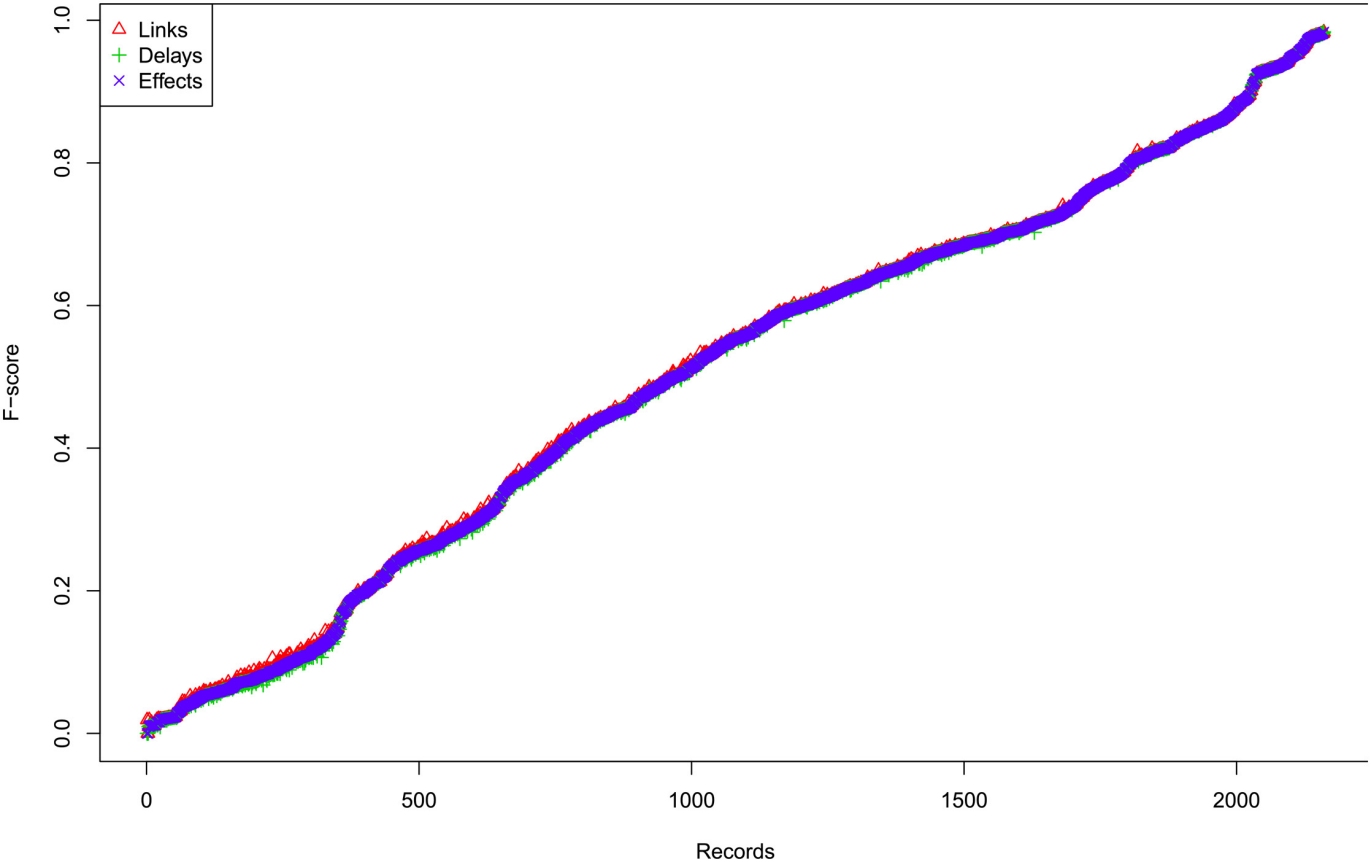
Profiles of F-scores of Links, Delays and Effects for Different Settings for Large Case. The x-axis shows the records.

**Fig 13:**
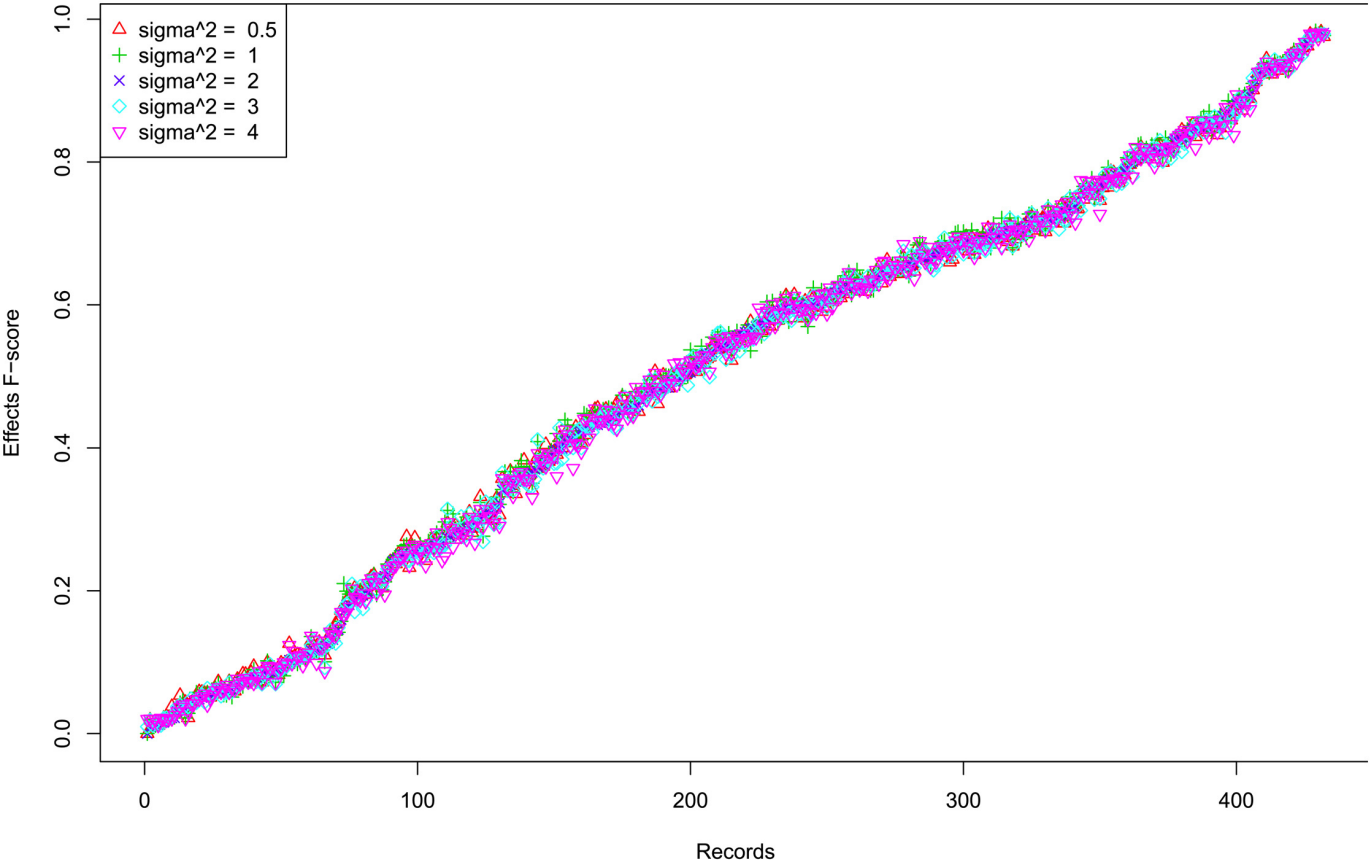
Profiles of Effects F-scores for Different *σ* ^2^ for Different Settings for Large Case. The x-axis shows the records.

Fig 12 shows the profiles of F-scores of *Links*, *Delays* and *Effects* for different settings for large case, which shows that the three are very consistent, so we omit the histograms of absolute differences here, but they are in Figures G to I in S1 File. In addition, Fig 13 shows the profiles of *Effects* F-scores for different *σ* ^2^ and Figure K in S1 File shows the corresponding boxplot for one segment case, and Figure N in S1 File for multiple segments case. Both show that for large case, the performance is very consistent for different *σ* ^2^. Therefore, in the following we show the results of *Effects* F-scores for *σ* ^2^ = 2 in Table 7 for one segment case, and in Table 8 for multiple segments case, where we show the performances of *complete*: CLINDE on the complete data (i.e. with also the expression of the “hidden” nodes), *hidden*: the proposed algorithm on the incomplete data (i.e. only the expression of observed genes), and *hiddenCL*: CLINDE on the incomplete data. Note that the *complete* case is mainly for comparison purpose, as in real-life, the hidden variables cannot be observed. It is unsurprising if the *complete* case has better performance, as CLINDE has more data in this case, so it is the comparison between *hidden* and *hiddenCL* that is of main interest here. The full results of median performance are shown in Table L in S1 File for one segment case and Table F in S1 File for multiple segments case. In Tables 7 and 8, we also show the ratio of F-score of *hidden* to that of *complete*, in percentages.

**Table 7:** Median Effects F-scores for One Segment Large Case with *σ* ^2^ = 2.

				complete (C)			hidden (H)			H/C			hiddenCL		
*n*	*n*_*h*_	*α*	*m*	st2	st3	st4	st2	st3	st4	st2	st3	st4	st2	st3	st4
50	5	0.5	20	0.123	0.088	0.021	0.097	0.055	0.009	78.7%	63.0%	45.0%	0.081	0.046	0.000
			50	0.413	0.397	0.279	0.323	0.269	0.171	78.3%	67.9%	61.4%	0.294	0.266	0.180
			100	0.659	0.667	0.608	0.532	0.517	0.446	80.7%	77.6%	73.5%	0.476	0.492	0.438
			200	0.788	0.847	0.815	0.656	0.715	0.667	83.3%	84.4%	81.8%	0.584	0.651	0.622
			400	0.847	0.933	0.933	0.737	0.808	0.816	87.1%	86.6%	87.5%	0.616	0.703	0.718
			800	0.850	0.966	0.979	0.769	0.878	0.893	90.5%	90.8%	91.3%	0.600	0.698	0.724
50	5	1	20	0.118	0.078	0.039	0.107	0.062	0.022	90.9%	78.8%	56.0%	0.078	0.054	0.021
			50	0.399	0.374	0.274	0.310	0.282	0.191	77.7%	75.5%	69.7%	0.288	0.275	0.183
			100	0.642	0.654	0.596	0.493	0.500	0.432	76.8%	76.5%	72.5%	0.481	0.500	0.438
			200	0.776	0.855	0.817	0.652	0.694	0.667	84.0%	81.2%	81.6%	0.592	0.655	0.632
			400	0.834	0.931	0.934	0.724	0.812	0.806	86.8%	87.2%	86.2%	0.616	0.711	0.719
			800	0.847	0.965	0.975	0.754	0.867	0.881	89.0%	89.9%	90.4%	0.603	0.705	0.730
50	5	2	20	0.136	0.120	0.066	0.116	0.104	0.054	85.2%	86.4%	81.7%	0.090	0.073	0.040
			50	0.385	0.384	0.300	0.298	0.284	0.210	77.5%	73.8%	70.0%	0.282	0.263	0.196
			100	0.607	0.626	0.575	0.453	0.457	0.409	74.6%	73.1%	71.2%	0.450	0.464	0.425
			200	0.765	0.831	0.806	0.601	0.646	0.626	78.5%	77.7%	77.6%	0.567	0.632	0.626
			400	0.820	0.932	0.933	0.663	0.760	0.762	80.9%	81.6%	81.7%	0.600	0.696	0.707
			800	0.833	0.959	0.976	0.731	0.850	0.870	87.8%	88.6%	89.1%	0.595	0.686	0.719
50	5	3	20	0.160	0.146	0.103	0.131	0.117	0.084	82.0%	80.2%	81.5%	0.107	0.086	0.058
			50	0.370	0.341	0.310	0.304	0.260	0.235	82.3%	76.3%	75.7%	0.263	0.246	0.220
			100	0.546	0.545	0.505	0.423	0.409	0.379	77.3%	75.1%	75.1%	0.396	0.399	0.377
			200	0.676	0.726	0.735	0.530	0.572	0.573	78.4%	78.9%	77.9%	0.505	0.558	0.569
			400	0.746	0.855	0.882	0.601	0.703	0.719	80.6%	82.3%	81.5%	0.554	0.648	0.688
			800	0.768	0.906	0.951	0.690	0.829	0.872	89.9%	91.4%	91.6%	0.558	0.668	0.706
100	10	0.5	20	0.071	0.060	0.016	0.075	0.047	0.011	106.6%	78.0%	68.9%	0.052	0.038	0.011
			50	0.347	0.364	0.281	0.259	0.252	0.181	74.8%	69.1%	64.3%	0.250	0.251	0.196
			100	0.597	0.654	0.602	0.453	0.495	0.435	75.8%	75.6%	72.2%	0.424	0.483	0.441
			200	0.720	0.833	0.819	0.595	0.687	0.676	82.6%	82.5%	82.6%	0.533	0.626	0.625
			400	0.781	0.925	0.933	0.674	0.798	0.813	86.3%	86.4%	87.2%	0.574	0.689	0.718
			800	0.773	0.948	0.977	0.700	0.865	0.892	90.5%	91.2%	91.3%	0.562	0.689	0.723
100	10	1	20	0.075	0.059	0.022	0.078	0.048	0.016	104.0%	81.9%	71.0%	0.051	0.037	0.012
			50	0.348	0.357	0.288	0.265	0.252	0.187	76.3%	70.7%	65.0%	0.255	0.251	0.194
			100	0.593	0.645	0.592	0.454	0.483	0.443	76.6%	74.9%	74.7%	0.431	0.477	0.432
			200	0.735	0.838	0.819	0.596	0.685	0.669	81.1%	81.8%	81.6%	0.545	0.627	0.628
			400	0.781	0.927	0.934	0.669	0.801	0.815	85.6%	86.4%	87.3%	0.567	0.683	0.703
			800	0.785	0.953	0.980	0.702	0.853	0.888	89.4%	89.6%	90.5%	0.557	0.684	0.721
100	10	2	20	0.106	0.095	0.061	0.106	0.084	0.041	99.3%	88.1%	68.2%	0.072	0.062	0.031
			50	0.350	0.366	0.315	0.255	0.259	0.218	72.6%	70.9%	69.3%	0.244	0.251	0.212
			100	0.563	0.618	0.587	0.414	0.454	0.422	73.5%	73.4%	71.9%	0.412	0.456	0.429
			200	0.702	0.814	0.817	0.542	0.641	0.630	77.2%	78.7%	77.1%	0.513	0.601	0.619
			400	0.761	0.913	0.937	0.628	0.760	0.784	82.6%	83.2%	83.7%	0.541	0.674	0.705
			800	0.771	0.947	0.981	0.686	0.856	0.887	89.0%	90.4%	90.5%	0.539	0.675	0.717
100	10	3	20	0.132	0.126	0.105	0.120	0.111	0.087	91.4%	88.1%	82.4%	0.088	0.079	0.061
			50	0.318	0.312	0.291	0.256	0.241	0.218	80.7%	77.3%	75.0%	0.224	0.211	0.188
			100	0.470	0.479	0.477	0.356	0.375	0.359	75.8%	78.3%	75.2%	0.341	0.355	0.353
			200	0.586	0.666	0.697	0.459	0.519	0.539	78.4%	78.0%	77.3%	0.441	0.501	0.531
			400	0.653	0.790	0.849	0.514	0.636	0.683	78.8%	80.5%	80.5%	0.484	0.597	0.651
			800	0.677	0.858	0.927	0.605	0.776	0.843	89.4%	90.4%	90.9%	0.484	0.618	0.688

The medians are taken over the 40 replicates. st2, st3 and st4 are for score thresholds of 2, 3 and 4 respectively. *complete* is using CLINDE on the complete data. *hidden* is our proposed algorithm on the incomplete data. *hiddenCL* is CLINDE on the incomplete data. H/C is the F-score of *hidden* divided by that of *complete* and shown as percentage.

**Table 8:** Median Effects F-scores for Multiple Segments Large Case with *σ* ^2^ = 2.

					complete (C)			hidden (H)			H/C			hiddenCL		
*n*	*n*_*h*_	*α*	*K*	*m*	st2	st3	st4	st2	st3	st4	st2	st3	st4	st2	st3	st4
50	5	0.5	1	30	0.213	0.175	0.094	0.163	0.119	0.063	76.8%	67.9%	67.2%	0.142	0.120	0.060
			2	59	0.449	0.453	0.341	0.339	0.307	0.206	75.5%	67.8%	60.3%	0.334	0.302	0.220
			4	100	0.622	0.633	0.558	0.488	0.467	0.400	78.4%	73.7%	71.7%	0.456	0.474	0.407
			8	196	0.770	0.816	0.768	0.623	0.647	0.614	80.9%	79.3%	80.0%	0.589	0.614	0.585
			16	400	0.867	0.922	0.905	0.733	0.782	0.775	84.6%	84.8%	85.7%	0.631	0.701	0.706
			32	812	0.887	0.968	0.968	0.754	0.846	0.865	85.1%	87.4%	89.4%	0.633	0.697	0.722
50	5	1	1	30	0.198	0.175	0.087	0.166	0.134	0.058	83.7%	76.3%	65.9%	0.150	0.127	0.051
			2	59	0.455	0.435	0.334	0.313	0.309	0.231	68.8%	71.1%	69.2%	0.329	0.306	0.234
			4	100	0.601	0.613	0.538	0.481	0.464	0.388	79.9%	75.8%	72.1%	0.458	0.461	0.386
			8	196	0.774	0.807	0.759	0.645	0.660	0.614	83.3%	81.8%	80.8%	0.584	0.611	0.590
			16	400	0.846	0.916	0.901	0.734	0.796	0.778	86.7%	87.0%	86.4%	0.645	0.694	0.697
			32	812	0.873	0.963	0.969	0.765	0.848	0.854	87.7%	88.1%	88.2%	0.631	0.707	0.717
50	5	2	1	30	0.199	0.167	0.096	0.146	0.118	0.058	73.6%	70.6%	60.3%	0.139	0.114	0.045
			2	59	0.428	0.417	0.333	0.331	0.296	0.241	77.5%	71.0%	72.4%	0.317	0.294	0.233
			4	100	0.600	0.621	0.547	0.479	0.467	0.403	79.8%	75.2%	73.7%	0.456	0.463	0.407
			8	196	0.777	0.809	0.779	0.619	0.658	0.614	79.6%	81.4%	78.8%	0.576	0.612	0.600
			16	400	0.851	0.925	0.914	0.714	0.782	0.769	83.9%	84.5%	84.2%	0.625	0.691	0.704
			32	812	0.876	0.966	0.972	0.736	0.822	0.831	84.0%	85.1%	85.5%	0.625	0.706	0.725
50	5	3	1	30	0.184	0.152	0.102	0.155	0.122	0.074	84.6%	80.2%	72.5%	0.130	0.104	0.062
			2	59	0.409	0.386	0.308	0.300	0.278	0.213	73.4%	71.9%	69.4%	0.293	0.285	0.210
			4	100	0.581	0.595	0.531	0.444	0.445	0.385	76.4%	74.8%	72.5%	0.424	0.438	0.393
			8	196	0.744	0.792	0.754	0.619	0.639	0.602	83.2%	80.7%	79.8%	0.567	0.611	0.582
			16	400	0.844	0.907	0.896	0.707	0.781	0.772	83.8%	86.1%	86.2%	0.629	0.698	0.695
			32	812	0.877	0.964	0.964	0.745	0.821	0.843	85.0%	85.1%	87.4%	0.628	0.703	0.716
100	10	0.5	1	21	0.071	0.058	0.021	0.069	0.051	0.016	97.9%	89.0%	75.1%	0.051	0.044	0.011
			2	48	0.307	0.299	0.233	0.232	0.213	0.160	75.5%	71.3%	68.8%	0.222	0.213	0.158
			4	92	0.530	0.567	0.518	0.398	0.420	0.364	75.0%	74.1%	70.2%	0.389	0.416	0.374
			8	188	0.719	0.789	0.758	0.574	0.625	0.606	79.7%	79.3%	80.0%	0.531	0.594	0.581
			16	387	0.808	0.910	0.902	0.678	0.772	0.769	83.9%	84.9%	85.2%	0.595	0.686	0.694
			32	811	0.831	0.960	0.974	0.720	0.844	0.860	86.7%	87.9%	88.3%	0.602	0.703	0.724
100	10	1	1	21	0.088	0.076	0.030	0.085	0.065	0.021	95.8%	85.1%	71.8%	0.060	0.057	0.020
			2	48	0.323	0.335	0.251	0.232	0.236	0.162	71.8%	70.5%	64.6%	0.222	0.223	0.158
			4	92	0.550	0.577	0.520	0.425	0.434	0.368	77.2%	75.3%	70.7%	0.399	0.426	0.367
			8	188	0.725	0.792	0.761	0.578	0.647	0.611	79.7%	81.7%	80.4%	0.539	0.600	0.580
			16	387	0.807	0.910	0.907	0.680	0.770	0.774	84.3%	84.6%	85.4%	0.603	0.685	0.692
			32	811	0.842	0.959	0.971	0.728	0.853	0.859	86.5%	89.0%	88.5%	0.607	0.698	0.721
100	10	2	1	21	0.083	0.067	0.036	0.086	0.055	0.029	103.2%	81.2%	80.3%	0.059	0.045	0.023
			2	48	0.296	0.295	0.249	0.221	0.214	0.164	74.6%	72.6%	65.8%	0.218	0.205	0.155
			4	92	0.529	0.559	0.518	0.404	0.422	0.382	76.3%	75.5%	73.7%	0.374	0.409	0.377
			8	188	0.711	0.780	0.761	0.578	0.626	0.602	81.4%	80.3%	79.1%	0.529	0.586	0.577
			16	387	0.806	0.914	0.906	0.669	0.755	0.759	83.1%	82.6%	83.7%	0.588	0.674	0.688
			32	811	0.836	0.959	0.977	0.715	0.839	0.857	85.4%	87.6%	87.7%	0.591	0.688	0.716
100	10	3	1	21	0.072	0.067	0.044	0.075	0.058	0.029	103.9%	87.1%	66.7%	0.048	0.044	0.026
			2	48	0.265	0.250	0.212	0.197	0.187	0.144	74.2%	74.9%	67.9%	0.189	0.177	0.141
			4	92	0.488	0.521	0.491	0.361	0.371	0.339	73.9%	71.1%	69.0%	0.348	0.372	0.347
			8	188	0.685	0.742	0.743	0.544	0.593	0.577	79.4%	79.9%	77.6%	0.497	0.554	0.562
			16	387	0.778	0.885	0.891	0.641	0.738	0.741	82.4%	83.4%	83.1%	0.565	0.660	0.685
			32	811	0.817	0.949	0.968	0.693	0.810	0.840	84.8%	85.4%	86.8%	0.578	0.691	0.717

The medians are taken over the 40 replicates. st2, st3 and st4 are for score thresholds of 2, 3 and 4 respectively. *K* is the number of segments used. *m* is the total number of time points of the segments used. *complete* is using CLINDE on the complete data. *hidden* is our proposed algorithm on the incomplete data. *hiddenCL* is CLINDE on the incomplete data. H/C is the F-score of *hidden* divided by that of *complete* and shown as percentage.

Generally, the F-score is better for larger *m*, however, note that even with *m* = 800 or *K* = 32 on the complete data, CLINDE cannot infer the whole GRN, because the GRNs are not small. For each of *complete*, *hidden* and *hiddenCL*, for *α* = 0.5,1 and 2, the F-scores are quite similar, though the F-scores are usually worse for *α* = 3 for one segment case; for multiple segments, the results are similar for *α* = 0.5,1,2, and 3. This shows the robustness of all three methods towards slight deviation from gaussianity for the error terms, though for larger deviation, the difference in performance is more noticeable. As for the score threshold, when *m* is small, smaller score threshold is better, and for larger *m*, larger score threshold is better.

When comparing between *complete*, *hidden* and *hiddenCL*, we see that in almost all settings, *complete* is better than *hidden*, which in turn is better than *hiddenCL*. The exceptions are all for *m* ≤ 100 or *K* ≤ 4. Note that *hidden*, using incomplete data, is usually able to achieve 70% to 80% of the performance of *complete*, and can get up to about 90% with large *m*. We have also performed one-sided Wilcoxon signed-rank test to test whether the median *Effects* F-score of *hidden* is better than *hiddenCL*. The p-values for *σ* ^2^ = 2 are shown in Table 9 for one segment case and in Table 10 for multiple segments case. And the p-values for other *σ* ^2^ are shown in Table C in S1 File for one segment case and in Table G in S1 File for multiple segments case. We see that for most settings, the p-values are very small, but occasionally the p-values are a bit larger for *m* ≤ 200 or *K* ≤ 4.

**Table 9:** P-values of one-sided Wilcoxon signed-rank test on whether the medians *Effects* F-scores of *hidden* is better than *hiddenCL* for the One Segment Large Case with *σ* ^2^ = 2.

*n*	*α*	*m*	st2	st3	st4
50	0.5	20	1.85082E-09	4.27927E-04	5.91254E-02
		50	1.22187E-06	1.97777E-02	7.05012E-01
		100	1.25890E-07	4.13188E-03	4.55023E-01
		200	9.09495E-13	6.36646E-12	5.21868E-09
		400	9.09495E-13	9.09495E-13	9.09495E-13
		800	9.09495E-13	9.09495E-13	9.09495E-13
50	1	20	3.64207E-08	1.03680E-04	1.36338E-02
		50	1.76024E-03	1.84770E-02	6.32721E-01
		100	4.21831E-04	1.47409E-01	9.18072E-01
		200	3.00133E-11	4.70440E-08	1.13534E-05
		400	9.09495E-13	9.09495E-13	1.81899E-12
		800	9.09495E-13	9.09495E-13	9.09495E-13
50	2	20	3.91992E-09	1.34587E-08	2.29484E-06
		50	1.78595E-04	1.03773E-02	4.49752E-03
		100	9.21563E-02	6.12502E-01	8.15900E-01
		200	3.38990E-06	5.88649E-03	1.47409E-01
		400	1.72804E-11	9.09495E-13	2.30102E-10
		800	9.09495E-13	9.09495E-13	9.09495E-13
50	3	20	4.06544E-10	9.09495E-13	1.92938E-07
		50	1.18116E-08	3.94010E-07	8.41174E-04
		100	2.84941E-04	2.22241E-03	1.62600E-01
		200	1.02952E-04	2.32674E-03	1.94996E-01
		400	1.27329E-11	6.36646E-12	1.18116E-08
		800	9.09495E-13	9.09495E-13	9.09495E-13
100	0.5	20	3.00133E-11	5.60030E-04	5.22645E-02
		50	3.95898E-03	1.84770E-02	9.97212E-01
		100	2.14964E-07	1.78595E-04	9.97084E-01
		200	9.09495E-13	1.81899E-12	5.82077E-10
		400	9.09495E-13	9.09495E-13	9.09495E-13
		800	9.09495E-13	9.09495E-13	9.09495E-13
100	1	20	1.81899E-12	3.23034E-08	1.06658E-01
		50	6.77883E-03	1.18525E-01	9.10652E-01
		100	1.33686E-06	2.78850E-03	4.39254E-01
		200	1.27329E-11	9.09495E-13	1.74014E-08
		400	9.09495E-13	9.09495E-13	9.09495E-13
		800	9.09495E-13	9.09495E-13	9.09495E-13
100	2	20	2.27374E-11	3.91083E-11	4.10137E-08
		50	3.13413E-05	2.84941E-04	1.31949E-03
		100	9.62277E-03	1.18525E-01	9.99385E-01
		200	6.00448E-09	3.03602E-07	6.83742E-04
		400	9.09495E-13	9.09495E-13	2.72848E-12
		800	9.09495E-13	9.09495E-13	1.85347E-08
100	3	20	9.09495E-13	6.36646E-12	9.09495E-13
		50	2.51475E-09	8.22183E-10	4.10137E-08
		100	5.05048E-05	1.22426E-05	5.76843E-03
		200	1.09601E-04	3.23034E-08	3.76574E-03
		400	1.26774E-07	6.36646E-11	4.87489E-10
		800	9.09495E-13	9.09495E-13	9.09495E-13

The tests are based on the 40 replicates. st2, st3 and st4 are for score thresholds of 2, 3 and 4 respectively.

**Table 10:** P-values of one-sided Wilcoxon signed-rank test on whether the medians *Effects* F-scores of *hidden* is better than *hiddenCL* for the Multiple Segments Large Case with *σ* ^2^ = 2.

*n*	*α*	*K*	*m*	st2	st3	st4
50	0.5	1	30	1.38532E-03	3.32451E-02	9.21645E-02
		2	59	1.97233E-02	6.95747E-01	7.61896E-01
		4	100	4.79873E-06	1.13695E-01	9.78903E-01
		8	196	8.00355E-11	5.81749E-08	5.81073E-07
		16	400	9.09495E-13	9.09495E-13	9.09495E-13
		32	812	9.09495E-13	9.09495E-13	9.09495E-13
50	1	1	30	6.77883E-03	6.05550E-03	1.17538E-01
		2	59	2.17906E-01	2.61704E-01	3.52344E-01
		4	100	6.10535E-06	2.18740E-02	1.56701E-01
		8	196	1.27329E-11	2.53194E-08	5.83590E-04
		16	400	9.09495E-13	9.09495E-13	2.72848E-12
		32	812	9.09495E-13	9.09495E-13	9.09495E-13
50	2	1	30	1.24866E-02	1.35190E-01	4.48704E-02
		2	59	6.00796E-03	4.18378E-01	3.05251E-01
		4	100	7.48913E-05	3.85003E-02	6.27698E-01
		8	196	1.00044E-10	3.56872E-07	1.68219E-04
		16	400	1.85347E-08	9.09495E-13	9.09495E-12
		32	812	9.09495E-13	9.09495E-13	9.09495E-13
50	3	1	30	1.01875E-06	1.28805E-04	1.59819E-03
		2	59	1.56904E-01	2.10106E-01	5.43996E-02
		4	100	3.19229E-04	5.09871E-03	7.05012E-01
		8	196	9.09495E-12	1.62785E-06	2.24322E-04
		16	400	9.09495E-13	9.09495E-13	9.09495E-13
		32	812	9.09495E-13	9.09495E-13	9.09495E-13
100	0.5	1	21	2.79215E-10	2.24432E-04	2.35524E-01
		2	48	1.05239E-05	1.60394E-02	6.91068E-01
		4	92	2.53916E-05	9.10447E-04	9.95689E-01
		8	188	9.09495E-13	6.36646E-12	1.93759E-07
		16	387	9.09495E-13	9.09495E-13	9.09495E-13
		32	811	9.09495E-13	9.09495E-13	9.09495E-13
100	1	1	21	2.30102E-10	7.64159E-07	4.52594E-03
		2	48	3.37733E-04	2.04941E-05	1.91553E-01
		4	92	9.13360E-08	4.69068E-03	3.42508E-01
		8	188	9.09495E-13	1.81899E-12	2.46640E-06
		16	387	9.09495E-13	9.09495E-13	9.09495E-13
		32	811	9.09495E-13	9.09495E-13	9.09495E-13
100	2	1	21	4.54747E-12	5.82077E-10	9.55109E-04
		2	48	2.78850E-03	1.13837E-03	4.20773E-02
		4	92	5.18348E-08	7.73253E-06	4.44501E-01
		8	188	9.09495E-13	9.09495E-13	3.04778E-06
		16	387	9.09495E-13	9.09495E-13	9.09495E-13
		32	811	9.09495E-13	9.09495E-13	9.09495E-13
100	3	1	21	1.81899E-12	5.37816E-07	5.09440E-07
		2	48	5.27871E-07	4.12623E-05	3.63203E-03
		4	92	3.99203E-04	3.57299E-01	8.94343E-01
		8	188	1.00044E-10	1.27329E-11	2.92082E-07
		16	387	9.09495E-13	9.09495E-13	9.09495E-13
		32	811	9.09495E-13	9.09495E-13	1.85347E-08

The tests are based on the 40 replicates. st2, st3 and st4 are for score thresholds of 2, 3 and 4 respectively. *K* is the number of segments used. *m* is the total number of time points of the segments used.

The results show the effectiveness of the proposed algorithm in detecting and estimating hidden nodes in large GRN, without knowing the number of hidden nodes and the variance of error terms.

### Heterogeneous Variance in Error Terms in Synthetic Large GRN

In previous sections, the error terms of all genes have the same constant variance in the synthetic data. This is an admittedly restrictive assumption. In this section, we test our algorithm on heterogeneous variance in the error terms for the synthetic large GRNs generated in the previous section, to test how robust HCC-CLINDE is towards violation of the assumption of constant variance in error terms. We simulate data as above, but instead of a constant, the variance of error terms for gene *i* is generated as $\sigma_{i}^{2}=\max\left(0.1,z_{i}\right)$, where *z*_*i*_ ∼ *N*(2,*δ* ^2^), and we test different values of *δ* ^2^:0.05, 0.1, 0.2, 0.5, 0.7, 0.9, 1.

#### Results

**Fig 14:**
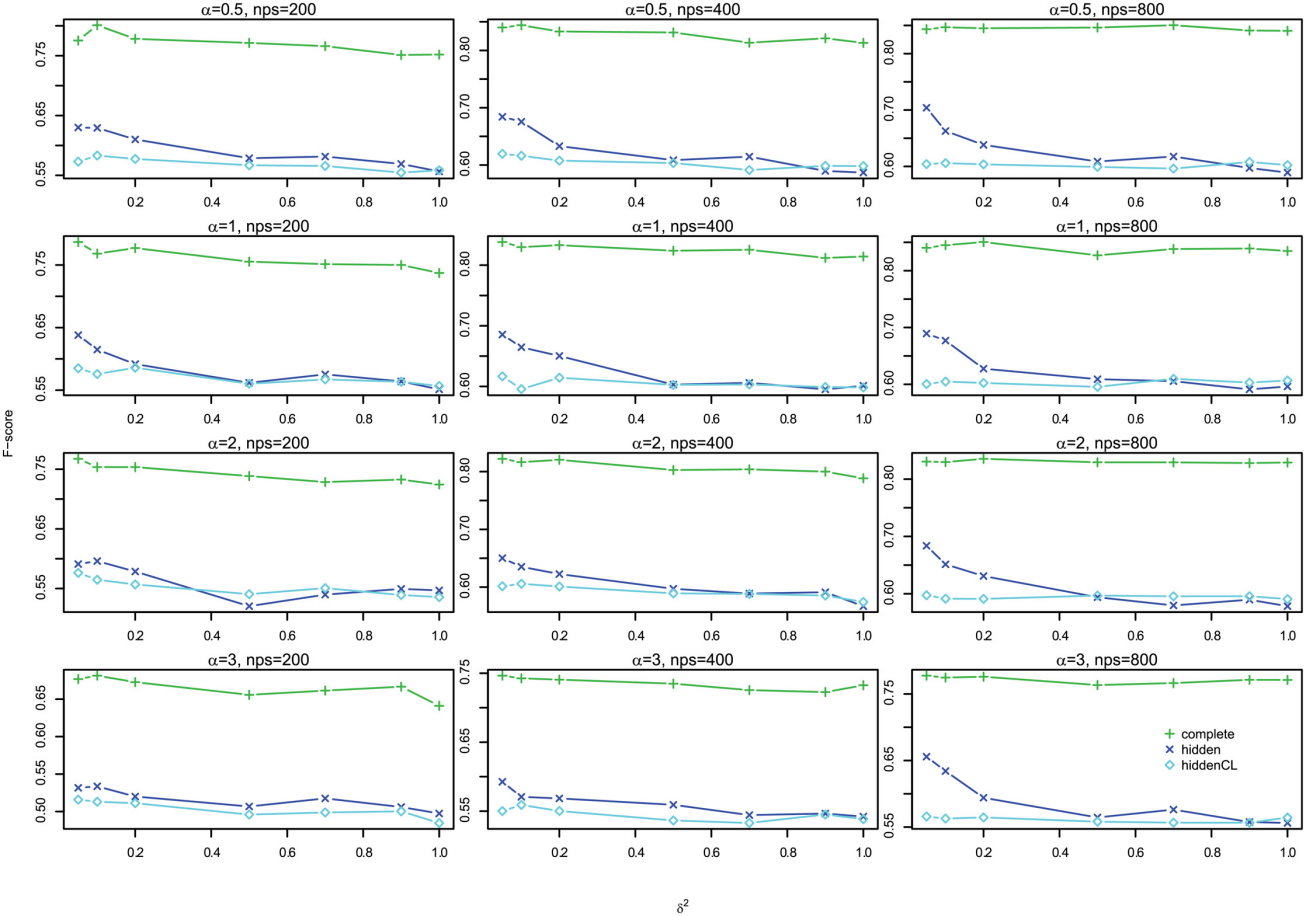
Median Effects F-scores for *n* = 50 with Different *δ* ^2^ for One Segment Large Case. *complete* is using CLINDE on the complete data. *hidden* is our proposed algorithm on the incomplete data. *hiddenCL* is CLINDE on the incomplete data. st used is 2.

**Fig 15:**
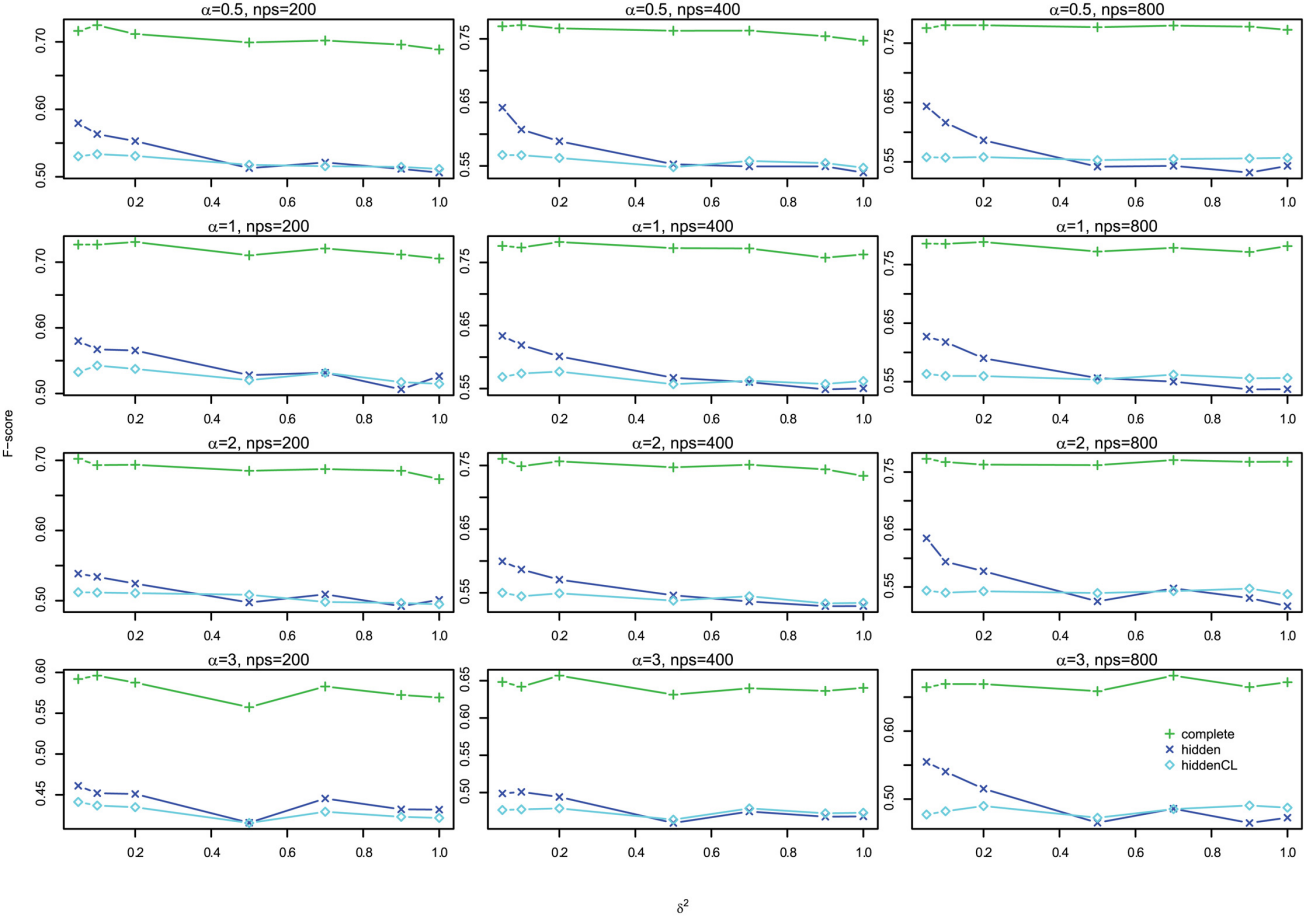
Median Effects F-scores for *n* = 100 with Different *δ* ^2^ for One Segment Large Case. *complete* is using CLINDE on the complete data. *hidden* is our proposed algorithm on the incomplete data. *hiddenCL* is CLINDE on the incomplete data. st used is 2.

**Fig 16:**
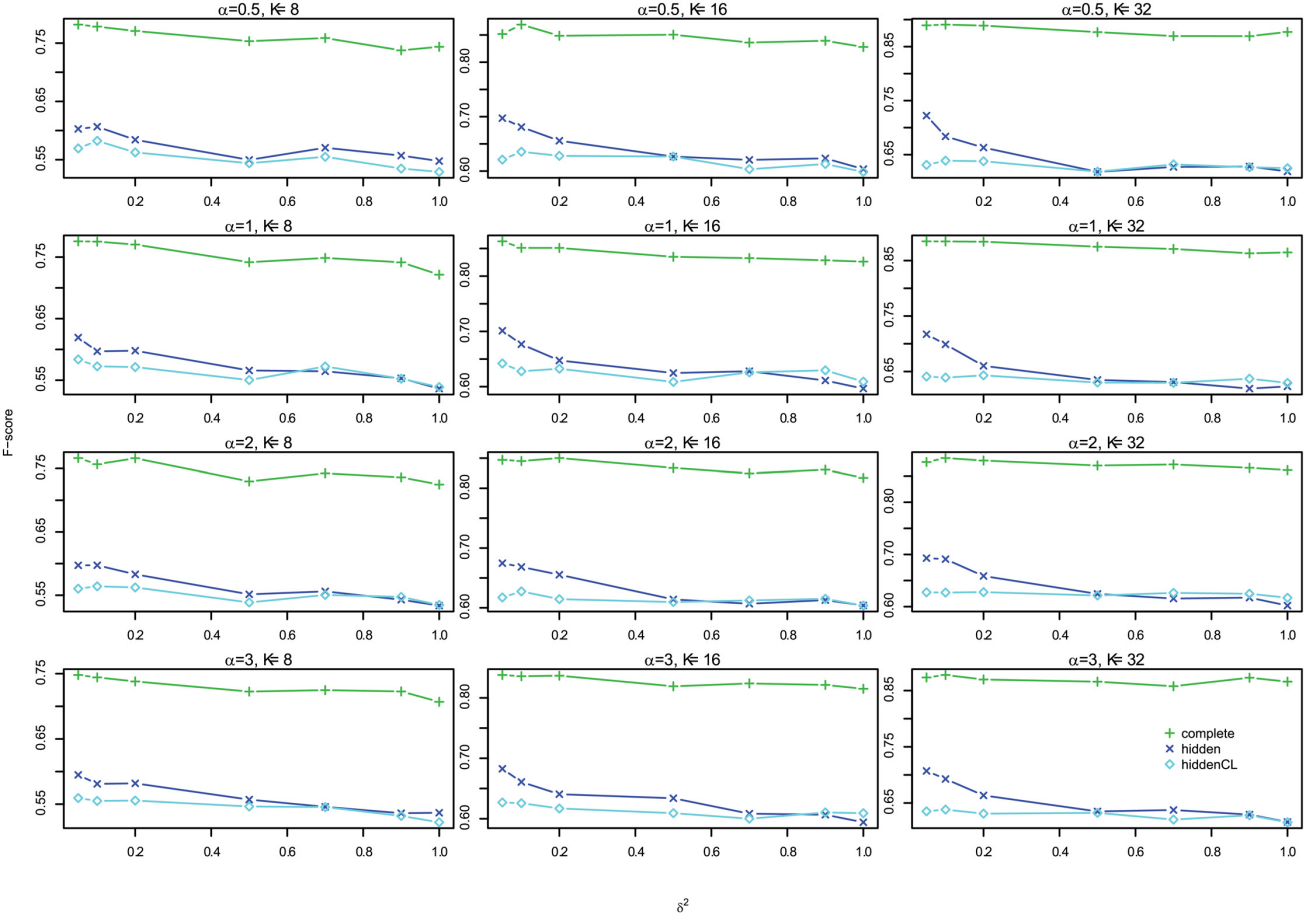
Median Effects F-scores for *n* = 50 with Different *δ* ^2^ for Multiple Segments Large Case. *complete* is using CLINDE on the complete data. *hidden* is our proposed algorithm on the incomplete data. *hiddenCL* is CLINDE on the incomplete data. st used is 2.

**Fig 17:**
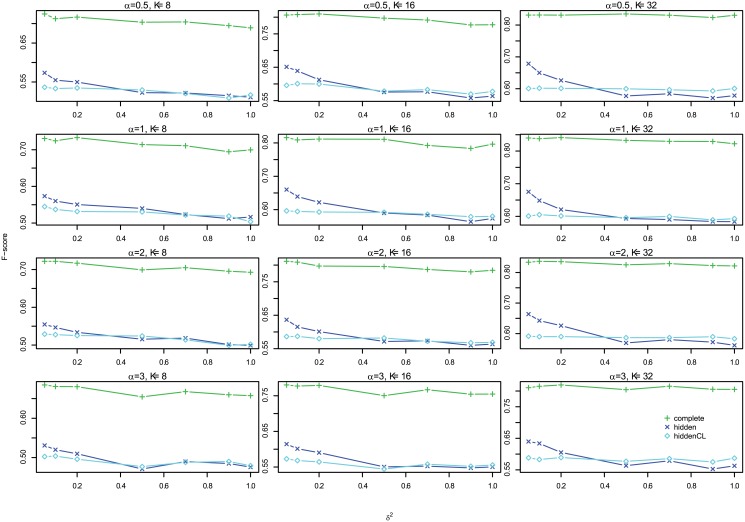
Median Effects F-scores for *n* = 100 with Different *δ* ^2^ for Multiple Segments Large Case. *complete* is using CLINDE on the complete data. *hidden* is our proposed algorithm on the incomplete data. *hiddenCL* is CLINDE on the incomplete data. st used is 2.

Figs 14 and 15 show the median *Effects* F-scores against different *δ* ^2^ for different *α* and different number of time points for one segment case with score threshold st = 2 for *n* = 50 and *n* = 100 respectively; while Figs 16 and 17 show the results for multiple segment case with st = 2 for *n* = 50 and *n* = 100 respectively. The full table of median F-scores (with different st, and also *Links* and *Delays* performances including *Recall*, *Precision* and *F-score*) are given in Tables H and I in S1 File for one segment case and multiple segments case respectively.

First, note that the median *Effects* F-scores for *complete* (which is CLINDE on the complete data) and *hiddenCL* (which is CLINDE on the incomplete data) are mostly stable for different values of *δ* ^2^, which is reasonable because CLINDE ignores hidden common cause, and makes no assumption on the variances of the error terms. We have also performed one-sided Wilcoxon signed-rank test to test whether the median *Effects* F-score of *hidden* is better than *hiddenCL*. The p-values are shown in Table 11 for st = 2 for both one segment and multiple segments cases, and the p-values for st = 3 and st = 4 are in Tables J and K in S1 File for one segment and multiple segments cases respectively. With sufficient time points (or segments), for small *δ* ^2^, the F-scores of *hidden* (which is HCC-CLINDE on the incomplete data) is smaller than *complete* but larger than *hiddenCL*, which is qualitatively the same as the constant variance case in previous section. Up to *δ* ^2^ = 0.2, the p-values are less than 0.001 for 400 or 800 time points, and 16 and 32 segments. As *δ* ^2^ increases, the F-scores decrease, and usually at *δ* ^2^ = 0.5, the performance of *hidden* would be close to *hiddenCL* and that *hidden* is no longer significantly better than *hiddenCL*, indicating that HCC-CLINDE cannot effectively recover the hidden common causes and has no advantage over ignoring hidden common causes. However, note that when *δ* ^2^ = 0.2, the variances of most genes are between 1 and 3, and when *δ* ^2^ = 0.5, most variances are between 0.1 and 4, which are moderately heterogeneous. Therefore, the results show that HCC-CLINDE can tolerate slight violation of the assumption of constant variances in the error terms of the genes.

**Table 11:** P-values of one-sided Wilcoxon signed-rank test on whether the medians *Effects* F-scores of *hidden* is better than *hiddenCL* for the Heterogeneous Variance Large Case.

*n*	*α*	*δ* ^2^	nps = 200	nps = 400	nps = 800	*K* = 8	*K* = 16	*K* = 32
50	0.5	0.05	**2.51475E-09**	**9.09495E-13**	**9.09495E-13**	**5.82077E-10**	**9.09495E-13**	**1.81899E-12**
		0.1	**1.46175E-06**	**1.72804E-11**	**1.53705E-10**	**2.92082E-07**	**3.91992E-09**	**6.36646E-11**
		0.2	**2.13304E-04**	**1.77279E-05**	**7.73253E-06**	**5.38597E-05**	**1.22187E-06**	**4.53190E-05**
		0.5	**2.68777E-02**	**3.51204E-02**	1.44876E-01	1.35193E-01	7.83223E-01	4.30922E-01
		0.7	2.25867E-01	**2.04007E-02**	**9.38085E-02**	1.66783E-01	1.84100E-01	3.48342E-01
		0.9	7.88755E-02	4.34017E-01	8.39846E-01	**2.78850E-03**	**8.50441E-02**	7.70093E-01
		1.0	3.72302E-01	9.06192E-01	8.22954E-01	1.84100E-01	8.60265E-01	9.08685E-01
50	1	0.05	**1.53705E-10**	**2.72848E-12**	**1.81899E-12**	**1.26774E-07**	**9.09495E-13**	**9.09495E-12**
		0.1	**1.85082E-09**	**3.00133E-11**	**1.72804E-11**	**3.17419E-06**	**1.00044E-10**	**4.06544E-10**
		0.2	**3.41121E-02**	**1.31954E-05**	**2.01109E-04**	**5.53261E-04**	**3.57201E-04**	**1.58393E-04**
		0.5	**4.91390E-02**	**2.96950E-02**	1.41598E-01	**4.35158E-02**	**2.59897E-02**	**6.03618E-02**
		0.7	**3.62532E-02**	2.90787E-01	5.02651E-01	1.91553E-01	2.66446E-01	7.89894E-01
		0.9	5.47211E-01	2.90403E-01	8.94343E-01	1.63447E-01	1.44329E-01	3.82076E-01
		1.0	1.73106E-01	7.05012E-01	8.08678E-01	4.30922E-01	9.73459E-01	7.82094E-01
50	2	0.05	**3.56872E-07**	**4.54747E-12**	**9.09495E-13**	**1.97442E-08**	**3.00133E-11**	**9.09495E-12**
		0.1	**1.68219E-04**	**1.74174E-06**	**2.86163E-08**	**8.23745E-06**	**7.30443E-08**	**3.91992E-09**
		0.2	**5.43599E-03**	**3.17871E-06**	**6.39177E-07**	**1.63188E-04**	**5.81749E-08**	**3.61960E-06**
		0.5	8.51795E-01	**1.66629E-02**	8.04618E-01	**2.86594E-02**	4.25285E-01	2.95777E-01
		0.7	6.07236E-01	3.97724E-01	9.67926E-01	2.72400E-01	6.47656E-01	9.75042E-01
		0.9	**8.93482E-02**	4.91650E-01	4.19823E-01	6.12502E-01	5.18552E-01	8.57140E-01
		1.0	1.19160E-01	9.80935E-01	9.98489E-01	2.59264E-01	4.92047E-01	9.99624E-01
50	3	0.05	**2.22241E-03**	**1.03501E-08**	**1.81899E-12**	**4.61341E-08**	**1.72804E-11**	**2.27374E-11**
		0.1	**6.83742E-04**	**2.84941E-04**	**1.24601E-10**	**3.35921E-05**	**5.81749E-08**	**2.51475E-09**
		0.2	5.52734E-01	**6.16046E-05**	**1.75435E-05**	**2.01897E-04**	**8.35792E-06**	**7.98702E-05**
		0.5	**1.00421E-03**	**4.09055E-02**	2.23464E-01	**1.79761E-03**	1.03308E-01	**8.68181E-02**
		0.7	**3.37733E-04**	1.23964E-01	**2.65413E-02**	2.17906E-01	1.38298E-01	**5.90745E-02**
		0.9	**1.78022E-02**	4.70862E-01	3.52344E-01	5.18552E-01	2.63607E-01	7.40736E-01
		1.0	**1.66100E-02**	2.42260E-01	9.90377E-01	5.44977E-01	5.50242E-01	9.77451E-01
100	0.5	0.05	**1.81899E-12**	**9.09495E-13**	**9.09495E-13**	**6.93035E-10**	**9.09495E-13**	**9.09495E-13**
		0.1	**1.72804E-11**	**9.09495E-13**	**2.72848E-12**	**1.53705E-10**	**4.54747E-12**	**9.09495E-13**
		0.2	**5.63730E-06**	**6.39177E-07**	**2.16005E-09**	**2.12223E-03**	**2.26382E-06**	**2.26382E-06**
		0.5	1.44329E-01	6.47656E-01	9.79599E-01	7.36393E-01	9.85053E-01	9.99799E-01
		0.7	5.13254E-01	9.90737E-01	9.94901E-01	4.81448E-01	9.91421E-01	9.98546E-01
		0.9	9.68908E-01	9.34221E-01	9.99999E-01	7.40736E-01	9.96814E-01	1.00000E+00
		1.0	9.61500E-01	9.96208E-01	9.99842E-01	9.62402E-01	9.99476E-01	9.99997E-01
100	1	0.05	**1.81899E-12**	**9.09495E-13**	**9.09495E-13**	**2.92221E-09**	**9.09495E-13**	**9.09495E-13**
		0.1	**5.21868E-09**	**9.09495E-12**	**6.36646E-11**	**2.63926E-07**	**8.22183E-10**	**1.53705E-10**
		0.2	**3.56872E-07**	**1.85082E-09**	**2.92082E-07**	**3.22983E-07**	**1.53150E-08**	**4.07638E-06**
		0.5	**1.44250E-02**	**8.71768E-02**	1.73583E-01	**1.66629E-02**	**5.59250E-02**	1.18525E-01
		0.7	6.17588E-01	6.37723E-01	9.93221E-01	2.94988E-01	8.12230E-01	9.63531E-01
		0.9	9.23105E-01	9.06192E-01	9.99988E-01	2.42260E-01	9.55396E-01	9.99643E-01
		1.0	1.41292E-01	9.94463E-01	9.99993E-01	**3.96647E-02**	7.76206E-01	9.99811E-01
100	2	0.05	**2.27374E-11**	**2.72848E-12**	**9.09495E-13**	**9.09495E-13**	**9.09495E-13**	**1.81899E-12**
		0.1	**3.75407E-06**	**1.00044E-10**	**3.00133E-11**	**5.20249E-06**	**6.36646E-11**	**1.81899E-12**
		0.2	**1.07730E-02**	**1.42158E-05**	**2.23736E-08**	**2.13304E-04**	**5.63730E-06**	**1.74014E-08**
		0.5	4.55023E-01	4.34017E-01	9.72496E-01	2.38104E-01	5.60747E-01	9.08441E-01
		0.7	**9.26339E-03**	9.59143E-01	7.45043E-01	**3.32894E-03**	2.21867E-01	9.45600E-01
		0.9	5.02651E-01	9.55396E-01	9.94463E-01	7.89894E-01	8.88969E-01	9.95689E-01
		1.0	4.39254E-01	9.10652E-01	9.99503E-01	4.28791E-01	8.70430E-01	9.99999E-01
100	3	0.05	**1.08313E-03**	**1.33686E-06**	**9.09495E-13**	**2.14964E-07**	**1.24601E-10**	**9.09495E-13**
		0.1	**1.39181E-02**	**6.10535E-06**	**1.81899E-12**	**1.57007E-07**	**4.06544E-10**	**2.27374E-11**
		0.2	**6.00796E-03**	**1.31954E-05**	**3.45537E-06**	**1.68219E-04**	**1.90650E-05**	**2.39727E-04**
		0.5	1.22987E-01	6.52587E-01	7.23150E-01	**5.20007E-02**	1.11031E-01	9.35002E-01
		0.7	**4.12623E-05**	7.82094E-01	9.83961E-01	5.50242E-01	9.21125E-01	9.59143E-01
		0.9	**5.76843E-03**	9.37642E-01	9.99993E-01	8.43097E-01	9.99554E-01	9.99989E-01
		1.0	1.84100E-01	7.53545E-01	9.99811E-01	8.29838E-01	9.81523E-01	1.00000E+00

The tests are based on the 40 replicates. st used is 2. *K* is the number of segments in multiple segment case. nps is the number of time points in single segment case.

### Small YEASTRACT Subnetworks with Real Data

#### Preprocessing of Subnetworks

We accessed YEASTRACT [55] (http://www.yeastract.com/formfindregulators.php) on 7th Feb, 2015 to get the regulating TFs of a list of 149 TFs. We chose “DNA binding and expression evidence” (which gives more confidence than having only binding evidence or expression evidence alone) and queried twice, once with “TF acting as activator” and once with “TF acting as inhibitor” to try to get the regulatory effects (positive or negative) of the regulatory relationships. The obtained 392 links involve only 129 TFs, and we used “ORF List ⇔ Gene List” utility of YEASTRACT (http://www.yeastract.com/formorftogene.php) to convert the gene names into ORF id’s, and all these 129 id’s appear in the yeast cell cycle [53] data.

However, the GRN is too large for the limited data, so we have chosen 22 subnetworks with sizes and constituent TFs shown in Table 12. For each subnetwork, a TF (which has children in the subnetwork) is chosen to be the hidden node. As the delays in the links are not known, and for a TF pair, some links may be both positive and negative in the YEASTRACT network, so we focus on the performance on *Links*.

**Table 12:** YEASTRACT Subnetworks.

sn	*n*	*n*_*L*_	Hidden TF	Other TFs
sn1	4	5	MBP1	ASH1, HCM1, SWI4
sn2	5	11	GLN3	DAL80, GAT1, GCN4, UGA3
sn3	6	5	ADR1	IME1, MSN4, PIP2, STE12, USV1
sn4	6	5	ASH1	ACE2, MBP1, SWI5, TOS8, YHP1
sn5	6	6	YAP6	CBF1, CIN5, MOT3, PDR1, TUP1
sn6	6	10	MSN2	ADR1, FHL1, NRG1, SOK2, USV1
sn7	6	12	DAL80	GAT1, GLN3, STE12, SUM1, TEC1
sn8	7	6	ACE2	ASH1, FKH2, GAT1, HMS2, INO4, SFL1
sn9	7	7	MET4	ABF1, HAP4, MET28, MET32, SFP1, TYE7
sn10	7	7	MSN4	ADR1, HAL9, RAP1, ROX1, RPN4, SOK2
sn11	7	7	UME6	GAT1, GSM1, LEU3, MSN2, OAF1, SIP4
sn12	7	8	STE12	MIG2, MSN2, PDR1, PDR3, SOK2, YAP1
sn13	7	9	CIN5	FLO8, IXR1, NRG1, XBP1, YAP1, YAP6
sn14	7	11	MCM1	MET32, STE12, SWI4, SWI5, TYE7, YAP3
sn15	7	11	RAP1	FKH1, FKH2, MCM1, SFP1, STE12, SWI5
sn16	7	14	FLO8	CIN5, HCM1, HMS1, STE12, TEC1, TOS8
sn17	9	12	PDR1	HAP4, MET28, PDR3, RPN4, SFL1, SWI4, YAP5, YAP6
sn18	9	16	RPN4	HSF1, MSN2, MSN4, PDR1, PDR3, PUT3, REB1, YAP1
sn19	10	17	STE12	CBF1, HAP4, MET4, MSN2, PDR1, RAP1, ROX1, SOK2, YAP1
sn20	11	13	ABF1	DAL81, ECM22, HAP1, HMS2, MET4, MGA1, REB1, RTG3, STP1, SUM1
sn21	12	23	STE12	ASH1, FLO8, OAF1, RAP1, RFX1, SFP1, SKO1, SOK2, TEC1, TOS8, XBP1
sn22	13	38	ROX1	FHL1, HAP1, HAP4, HMS1, IXR1, MSN2, MSN4, SKN7, SKO1, STE12, XBP1, YAP1

*sn* is the subnetwork. *n* is the number of TFs in the subnetwork, *n*_*L*_ is the number of links in the subnetwork. The hidden TF is the one with expression hidden in *incomplete* setting of the experiments.

#### Preprocessing of Expression Data

We downloaded the yeast cell cycle [53] data from http://genome-www.stanford.edu/cellcycle/. The expression data contains 4 time series: *alpha*, *cdc15*, *cdc28* and *elu*, with different lengths and time points, as shown in the second column of Table 1. Since the time steps of the 4 time series are not all the same, for each TF, we perform spline interpolation (using the spline() function in R) to the time points shown in the third column of Table 1. Also, some TFs in some series are entirely missing (this seems more often in *cdc15*), and we fill in with zero. For other missing values, we rely on the spline interpolation to fill in the value.

After this, for each subnetwork we use the converted ORF id of each TF to retrieve expression data of the TFs on the 4 time series, to obtain one set labeled *complete* which contains the expression data of all TFs of the subnetwork, and another labeled *incomplete* which contains the expression data of all but the chosen TF of the subnetwork. Therefore, for each subnetwork, we have 8 expression datasets.

#### Results

The *Links* F-scores of CLINDE with *complete* data, our proposed algorithm (with normalization of data, with unknown *σ* ^2^ and unknown number of hidden nodes) on *incomplete* data, and CLINDE on *incomplete* data on the subnetworks are shown in Table 13, where we set the maximum delay *τ*_0_ to be 4, and use the score threshold of 1 as the number of time points is not large. We run the algorithms (ours and CLINDE) separately on one time series and used the 4 time series together (*all*), because both CLINDE and our proposed algorithm can handle multiple segments of time series. We show which segment(s) have the best performance.

**Table 13:** Links F-scores for YEASTRACT Subnetworks.

			complete (C)		hidden (H)		H/C	hiddenCL	
sn	*n*	*n*_*L*_	best	which	best	which	best	best	which
sn1	4	5	**0.889**	cdc28	0.444	cdc28, elu	50.0%	0.364	alpha, cdc28
sn2	5	11	**0.476**	cdc15	0.324	all, alpha	68.1%	0.267	alpha
sn3	6	5	0.286	cdc28	**0.364**	cdc15	**127.3%**	0.000	—
sn4	6	5	**0.333**	alpha	**0.333**	cdc15	100.0%	0.000	—
sn5	6	6	**0.308**	alpha	**0.308**	cdc28	100.0%	0.000	—
sn6	6	10	**0.646**	cdc28	0.545	elu	84.4%	0.471	elu
sn7	6	12	0.526	cdc15	**0.556**	elu	**105.6%**	0.455	cdc15
sn8	7	6	0.286	cdc28	**0.333**	elu	**116.7%**	0.000	—
sn9	7	7	**0.429**	cdc28	0.375	cdc15	87.5%	0.154	cdc28
sn10	7	7	0.293	cdc28	**0.353**	cdc15	**120.6%**	0.000	—
sn11	7	7	**0.353**	cdc28	**0.353**	alpha	100.0%	0.133	cdc28
sn12	7	8	0.421	elu	0.444	cdc15	**105.6%**	**0.500**	cdc15
sn13	7	9	0.305	all	**0.375**	alpha	**122.8%**	0.083	all
sn14	7	11	0.381	cdc28	**0.421**	cdc28	**110.5%**	0.174	cdc15
sn15	7	11	0.320	elu	**0.385**	all	**120.2%**	0.245	cdc15, cdc28
sn16	7	14	0.441	elu	**0.545**	cdc15	**123.6%**	0.417	elu
sn17	9	12	0.296	elu	**0.364**	alpha	**122.7%**	0.174	elu
sn18	9	16	0.214	cdc15	**0.367**	cdc15	**171.3%**	0.154	cdc15
sn19	10	17	0.253	cdc28	**0.333**	cdc15	**131.9%**	0.253	cdc28
sn20	11	13	0.282	cdc15	**0.435**	alpha	**154.3%**	0.000	—
sn21	12	23	**0.386**	elu	0.326	elu	84.5%	0.295	elu
sn22	13	38	0.190	elu	**0.309**	cdc15	**162.2%**	0.136	elu

*sn* is the subnetwork. The score threshold is 1. *n* is the number of TFs in the subnetwork, *n*_*L*_ is the number of links in the subnetwork. *complete* is using CLINDE on the complete data. *hidden* is our proposed algorithm on the incomplete data. *hiddenCL* is CLINDE on the incomplete data. H/C is the F-score of *hidden* divided by that of *complete* and shown as percentage. *best* is the best of the four segments, and *which* shows the best segment (*all* is using all 4 segments). The maximum delay *τ*_0_ used is 4.

First, as the 4 time series segments are from different experimental conditions, so it is possible that different genes have better responses in different segments. Consequently, using all 4 segments (*all*) may not give the best results, even though the total number of time points is larger.

Second, our proposed algorithm on incomplete data has better F-scores than CLINDE on complete data in 14 out of 22 subnetworks, with 3 ties. There are two possible reasons. One is that after estimating the initial GRN, the subsequent steps make some assumptions on the structure of the GRN around the hidden common cause(s). This may help the GRN inference, especially in case of limited data. The other is that after estimating the hidden common cause(s), the re-estimation of the links around the hidden common cause(s) works only on a subnetwork. However, we expect that as the amount of (quality) data increases, the situation would be more like the synthetic large case, where CLINDE on complete data has better performance, but our algorithm is close to it.

Third, our proposed algorithm on incomplete data outperforms CLINDE on incomplete data in 21 out of 22 subnetworks. This is as expected, as CLINDE is unaware of the presence of hidden node(s), so there could be misleading links (as illustrated in Fig 1).

In short, due to limited real expression data, we cannot draw very strong conclusions, but the results indicate the potential of our proposed algorithm in recovering hidden common cause(s) using real data, when the error variance and the number of hidden node(s) is unknown.

## Discussions

In this section, we discuss possible extensions to the basic algorithm introduced above to relax some assumptions made.

### Variance of Error Terms

In the proposed algorithm, we assume that the variance of the error terms is a constant. This assumption is used in detection of genes with hidden common cause, and in calculation of correlation threshold in clustering.

This assumption can be relaxed, for example to assume that the error terms of some genes have variance $\sigma_{1}^{2}$ and some have variance $\sigma_{2}^{2}$. After inferring an initial GRN and calculating the empirical variances of the error terms, instead of using the median as estimate of the true variance, we may cluster the empirical variances and use the centers of the two largest clusters as estimates of $\sigma_{1}^{2}$ and $\sigma_{2}^{2}$. Having obtained the estimates (or assumed given if the number of observed genes is too small to allow good estimation), those observed genes with empirical variances too far away from the estimates are predicted to have hidden common cause.

The variance of the error terms is also used in calculating the threshold of absolute correlation in clustering the genes having the same hidden common cause. If we assume more than one possible variance, we may use the larger one to calculate a conservative threshold. Alternatively, for each observed gene, we may choose the estimate closer to the empirical variance as its true variance, and calculate the threshold accordingly. At this point, it is unclear which method gives better clustering, and further study is needed.

However, it is difficult to handle a very general and spread-out distribution of the variances of the error terms. If the distribution is unknown and we desire to estimate it from the empirical variances, a large number of observed genes is needed for meaningful estimation, but this increases the difficulty of inference of the initial GRN substantially because of the increased number of genes. Even if we assume the distribution is known or well estimated, given an empirical variance, we need to decide whether this is *as expected*, which is essentially a statistical test, which is more difficult for less concentrated distribution.

### Discrete Data

The proposed algorithm handles continuous data, here we discuss the possibility of extending it to discrete time series data. For this purpose, a few parts would need to be adapted.

Firstly, the data model would be different. Instead of a linear combination of its parents’ values (with time delay) plus an additive error, the value of a node would depend on its parents through a conditional distribution. In addition, we assume that given each configuration of the parents, the conditional distribution has most probability (e.g. 1−*p*_*e*_) concentrated on a value, and the remaining *p*_*e*_ is spread over other values, i.e. the value of the node is roughly a function of its parents, with a “noise level” of *p*_*e*_.

Secondly, the inference of initial GRN needs adaptation. This is simple because similar to the PC algorithm [29], CLINDE can use any type of independence and conditional independence test suitable for the data. For example, *χ* ^2^ tests could be used instead of the correlation test.

Thirdly, we need to predict the nodes with hidden common cause based on the initial GRN. Given an initial GRN, we could estimate the maximum likelihood conditional distribution of each node from the data, and analogous to variance of the error terms, the empirical “noise level” could be calculated and compared with the expected level to predict whether that node has hidden common cause. If we assume a constant “noise level” for all the nodes, it could be given (for small number of nodes) or estimated from empirical “noise levels” (for large number of nodes).

Fourthly, for the clustering of nodes having the same hidden common cause, since two nodes having the same parent would be associated, so it is only necessary to determine an appropriate threshold for the association measure based on the “noise levels”.

Lastly, instead of using Singular Value Decomposition to estimate hidden common cause(s), a different method has to be used. For example, Structural-EM could be used as in [51], and the method in [52] could be used for the related problem of determining the number of states for the hidden common cause(s).

### Relax Structural Assumptions

#### Nodes with More than One Hidden Common Cause

In the basic algorithm, we assume that if a gene has a hidden parent, it has no other parents. Here we consider the possibility that a gene has two or more hidden parents (both hidden common causes). This should pose little difficulty for predicting the genes with hidden common cause(s), as they would have the wrong parent(s) in the initial GRN, and consequently the empirical variance of they error terms are likely different from expected. On the other hand, the clustering and estimation of hidden common causes would need some adaptation. For clustering, the determination of the correlation threshold is not as straightforward. One simple strategy is to use a fixed conservative threshold, and hope that genes without the same hidden common cause(s) would not be close enough to have high correlation. And for the estimation of the hidden common cause(s), we may still apply SVD, but use more singular vectors corresponding to the largest singular values. The main difficulty is that a way is needed to determine how many singular vectors to take, i.e. how many hidden common causes the set of genes have. One possible strategy is to successively take more singular vectors, until the drop in residual error is small. But this is a sort of model selection problem, which is not straightforward. Therefore, more study is needed to determine whether the strategy would work well.

#### Multiple Layers of Hidden Nodes

The current model and algorithms assumes that any hidden common cause does not have other hidden common cause as parents, i.e. the hidden common causes are not connected directly to each other. This simplifying assumption may be restrictive in some cases, where multiple layers of hidden common causes may exist and may have meaningful interpretation. One possible strategy is to first infer possible hidden common cause(s) of observed genes, then treat them as observed (because our algorithm also estimates the time series of the predicted hidden common cause(s)), and repeat the process to see if further hidden common cause(s) could be inferred. However, at this stage, it is unclear whether this strategy would work well, because the estimated time series of the hidden common cause(s) may not be accurate enough. Moreover, it is unclear that whether it is even feasible to infer rich structures among hidden nodes without prior knowledge or assumptions, given only limited data of observed nodes. We leave these issues as future work.

### Relative Error Model

The proposed algorithm assumes a constant variance for the additive error terms, and the variance could be known or estimated from empirical variances. Alternatively, we may assume a model where the variance of the error terms is a constant proportion of the variance of the gene. This could be easily handled by (centering and) normalizing the input expression data such that each gene has a variance of one (except those with constant expression). The proportion of variance of error terms to the variance of the gene would remain unchanged, but now it is also the variance of the error terms. This normalization does not affect the inference of the initial GRN, as CLINDE uses correlation and conditional correlation tests, which are unaffected by centering and scaling.

### Delays with Distribution

In our current model, we assume the delay between two genes is deterministic (but unknown and is to be found). While this assumption makes the model and algorithm simpler, in reality, due to the stochastic operations of the cell, it is more realistic to model the delays as random variable, e.g. with exponential distribution as in [32] and [33]. We leave this as future work.

### Not Using Time Series Data

The basic algorithm uses time series data, here we briefly consider the possibility of using non-time series data. To begin with, without time series data, it would be impossible to estimate the delays in the links, but it may still be possible to infer the directions of the links, just as in the PC algorithm [29]. However, without time series data, inferring the directions of the links is much more difficult, especially when we allow the presence of cycles in the causal network. Also, the directions of some links may still remain undetermined given the data, because both directions are consistent with the data. The clustering and estimation of hidden common cause, on the other hand, does not pose great difficulty when using non-time series data, assuming the initial GRN is well estimated.

## Conclusion

To summarize, we have developed an algorithm to recover the causal GRN with delays in the regulatory links from time series expression data, where a small but unknown number of nodes are hidden, i.e. without expression data. We have tested our algorithm on 3 types of synthetic data: small GRN with one hidden node, small GRN with no hidden node, and large GRN with a small but unknown number of hidden nodes. Results on synthetic data show that our algorithm can effectively recover the causal GRN. We have also demonstrated our algorithm on small subnetworks of YEASTRACT with real expression data, and the results show the potential of our algorithm to recover hidden nodes using real data.

## Supporting Information

S1 File Containing various supplementary figures and tables, listed as follows. **Figure A, Histogram of Absolute Differences of F-scores of Delays and Effects for One Segment Small Hidden Case. Figure B, Histogram of Absolute Differences of F-scores of Links and Delays for One Segment Small Hidden Case. Figure C, Profiles of F-scores of Links, Delays and Effects for Different Settings for One Segment Small Non-Hidden Case. Figure D, Histogram of Absolute Differences of F-scores of Links and Effects for One Segment Small Non-Hidden Case. Figure E, Histogram of Absolute Differences of F-scores of Delays and Effects for One Segment Small Non-Hidden Case. Figure F, Histogram of Absolute Differences of F-scores of Links and Delays for One Segment Small Non-Hidden Case. Figure G, Histogram of Absolute Differences of F-scores of Links and Effects for One Segment Large Case. Figure H, Histogram of Absolute Differences of F-scores of Delays and Effects for One Segment Large Case. Figure I, Histogram of Absolute Differences of F-scores of Links and Delays for One Segment Large Case. Figure J, Boxplot of Effects F-score with Different *σ*^2^ for One Segment Small Non-Hidden Case. Figure K, Boxplot of Effects F-score with Different *σ*^2^ for One Segment Large Case. Figure L, Boxplot of Effects F-score with Different *σ*^2^ for Multiple Segments Small Hidden Case. Figure M, Boxplot of Effects F-score with Different *σ*^2^ for Multiple Segments Small Non-Hidden Case. Figure N, Boxplot of Effects F-score with Different *σ*^2^ for Multiple Segments Large Case. Table A, Full Results of Median Performance for One Segment Small GRN with One Hidden Node.** e2 is *σ* ^2^, alpha is *α*, nps is number of time points *m*, st is the score threshold. For the data column, hidden means using our proposed algorithm on the incomplete data, complete means using CLINDE on the complete data (i.e. all nodes are not hidden), hiddenCL means using CLINDE on the incomplete data. l2., d2. and e2. are *Links*, *Delays* and *Effects* respectively. r is *Recall*, p is *Precision* and f is *F-score*. Each median is taken over 20 replicates. **Table B, Full Results of Median Performance for One Segment Small GRN without Hidden Node.** e2 is *σ* ^2^, alpha is *α*, nps is number of time points *m*, st is the score threshold. For the data column, hidden means using our proposed algorithm on the incomplete data. l2., d2. and e2. are *Links*, *Delays* and *Effects* respectively. r is *Recall*, p is *Precision* and f is *F-score*. Each median is taken over 20 replicates. **Table C, P-values of one-sided Wilcoxon signed-rank test on whether the medians *Effects* F-scores of *hidden* is better than *hiddenCL* for the One Segment Large Case. Table D, Full Results of Median Performance for Multiple Segments Small GRN with One Hidden Node.** e2 is *σ* ^2^, alpha is *α*, nsegs is number of segments *K*, st is the score threshold. For the data column, hidden means using our proposed algorithm on the incomplete data, complete means using CLINDE on the complete data (i.e. all nodes are not hidden), hiddenCL means using CLINDE on the incomplete data. l2., d2. and e2. are *Links*, *Delays* and *Effects* respectively. r is *Recall*, p is *Precision* and f is *F-score*. Each median is taken over 20 replicates. **Table E, Full Results of Median Performance for Multiple Segments Small GRN without Hidden Node.** e2 is *σ* ^2^, alpha is *α*, nsegs is number of segments *K*, st is the score threshold. For the data column, hidden means using our proposed algorithm on the incomplete data. l2., d2. and e2. are *Links*, *Delays* and *Effects* respectively. r is *Recall*, p is *Precision* and f is *F-score*. Each median is taken over 20 replicates. **Table F, Full Results of Median Performance for Multiple Segments Large GRN with More than One Hidden Node with** *n* = 50 **and** *n* = 100. ng is the number of observed genes *n*, nh is the number of hidden nodes *n*_*h*_, e2 is *σ* ^2^, alpha is *α*, nsegs is number of segments *K*, st is the score threshold. For the data column, hidden means using our proposed algorithm on the incomplete data, complete means using CLINDE on the complete data (i.e. all nodes are not hidden), hiddenCL means using CLINDE on the incomplete data. l2., d2. and e2. are *Links*, *Delays* and *Effects* respectively. r is *Recall*, p is *Precision* and f is *F-score*. Each median is taken over 40 replicates. **Table G, P-values of one-sided Wilcoxon signed-rank test on whether the medians *Effects* F-scores of *hidden* is better than *hiddenCL* for the Multiple Segments Large Case. Table H, Full Results of Median Performance for One Segments Large GRN with Different** *δ* ^2^ **with** *n* = 50 **and** *n* = 100. ng is the number of observed genes *n*, nh is the number of hidden nodes *n*_*h*_, e2 is *σ* ^2^, alpha is *α*, nps is number of time points *m*, st is the score threshold. For the data column, hidden means using our proposed algorithm on the incomplete data, complete means using CLINDE on the complete data (i.e. all nodes are not hidden), hiddenCL means using CLINDE on the incomplete data. l2., d2. and e2. are *Links*, *Delays* and *Effects* respectively. r is *Recall*, p is *Precision* and f is *F-score*. d2 is *δ* ^2^. Each median is taken over 40 replicates. **Table I, Full Results of Median Performance for Multiple Segments Large GRN with More than One Hidden Node with** *n* = 50 **and** *n* = 100. ng is the number of observed genes *n*, nh is the number of hidden nodes *n*_*h*_, e2 is *σ* ^2^, alpha is *α*, nsegs is number of segments *K*, st is the score threshold. For the data column, hidden means using our proposed algorithm on the incomplete data, complete means using CLINDE on the complete data (i.e. all nodes are not hidden), hiddenCL means using CLINDE on the incomplete data. l2., d2. and e2. are *Links*, *Delays* and *Effects* respectively. r is *Recall*, p is *Precision* and f is *F-score*. d2 is *δ* ^2^. Each median is taken over 40 replicates. **Table J, P-values of one-sided Wilcoxon signed-rank test on whether the medians *Effects* F-scores of *hidden* is better than *hiddenCL* for the Heterogeneous Variance One Segment Large Case. Table K, P-values of one-sided Wilcoxon signed-rank test on whether the medians *Effects* F-scores of *hidden* is better than *hiddenCL* for the Heterogeneous Variance Multiple Segments Large Case. Table L, Full Results of Median Performance for One Segment Large GRN with More than One Hidden Node with** *n* = 50 **and** *n* = 100. ng is the number of observed genes *n*, nh is the number of hidden nodes *n*_*h*_, e2 is *σ* ^2^, alpha is *α*, nps is number of time points *m*, st is the score threshold. For the data column, hidden means using our proposed algorithm on the incomplete data, complete means using CLINDE on the complete data (i.e. all nodes are not hidden), hiddenCL means using CLINDE on the incomplete data. l2., d2. and e2. are *Links*, *Delays* and *Effects* respectively. r is *Recall*, p is *Precision* and f is *F-score*. Each median is taken over 40 replicates.
(ZIP)
